# Identification of potential vinorelbine-associated prognostic genes in breast cancer through integrative bioinformatics and experimental validation

**DOI:** 10.3389/fonc.2026.1855523

**Published:** 2026-06-26

**Authors:** Yi Wu, Guimei Yang, Yixian Li, Yunjing Ruan, Qianmei Yang

**Affiliations:** 1 Experiment Center for Medical Science Research, Kunming Medical University, Kunming, Yunnan, China; 2 School of Pharmaceutical Science & Yunnan Provincial Key Laboratory of Pharmacology for Natural Products, Kunming Medical University, Kunming, Yunnan, China

**Keywords:** breast cancer, vinorelbine-related genes, random survival forest model, immune microenvironment, single-cell analysis

## Abstract

**Background:**

The mechanisms underlying the occurrence and development of breast cancer (BC) is complex. Vinorelbine-related genes (Vino-RGs) may play important roles in the treatment of BC, but their specific mechanisms remain unclear. We aimed to explore vinorelbine-related prognostic genes and their mechanisms for BC treatment.

**Methods:**

BC-related data were collected from public databases. Differentially expressed genes (DEGs) were screened based on TCGA database, and candidate genes were gained by intersecting with Vino-RGs. Prognostic genes were determined using Cox regression and machine learning algorithms. A random survival forest (RSF) model was built and evaluated, followed by construction and evaluation of a nomogram. Enrichment analysis, and immune microenvironment analysis were carried out. Single-cell analysis was used to further explore the development mechanism of BC. Finally, RT-qPCR was conducted to explore the expression of prognostic genes.

**Results:**

TUBA1C, BRCA1, TGFB1, TUBA1B, XRCC1, PTGS2, IL7, and TUBB2B were screened as prognostic genes for BC. The constructed RSF model and nomogram showed good predictive accuracy for the prognosis of BC patients. Multiple pathways related to BC progression were identified. Immune microenvironment analysis revealed the correlations between the risk score and immune cells and the poor prognosis for BC patients with lower tumor microenvironment (TME) scores and immune phenotype score (IPS) in high risk group (HRG). AZD1332_1463, BMS.754807_2171, mitoxantrone_1810, and nutlin.3a…._1047 had significantly positive correlations with the risk score. Macrophages were identified as crucial cells for BC development. Macrophages had active communication with other cells, and the expression of prognostic genes differed in the whole differentiation of macrophages. RT-qPCR analysis revealed significantly higher expressions of TGFB1 and BRCA1 in BC samples compared to BC-VLB+PD samples, while IL7 and PTGS2 expressions were significantly lower in BC samples.

**Conclusion:**

This study identified TUBA1C, BRCA1, TGFB1, TUBA1B, XRCC1, PTGS2, IL7, and TUBB2B as prognostic genes for breast cancer that are potentially associated with vinorelbine treatment, offering actionable biomarkers for individualized prognosis assessment and guiding patient stratification for vinorelbine-based combination therapies in clinical practice.

## 1 Introduction

Breast cancer (BC) is the predominantly malignancy among women and a major reason for cancer-related mortality worldwide. Despite advances in early detection and multimodal treatment strategies, including surgery, chemotherapy, radiotherapy, endocrine therapy, and immunotherapy, disease recurrence and metastasis continue to pose major clinical challenges (1, 2). This unfavorable outcome is largely attributable to the pronounced heterogeneity of BC at the molecular, cellular, and microenvironmental levels. Although numerous prognostic genes and gene signatures have been identified and applied to guide cancer management, many of these markers were originally developed in other solid tumors and demonstrate limited predictive value when directly applied to BC cohorts, highlighting the necessity for identifying more robust and disease-specific prognostic indicators for BC (3, 4).

Recent studies have emphasized that the prognostic relevance of tumor-associated genes is closely linked to the immune microenvironment and treatment responsiveness in BC. Large-scale transcriptomic analyses have revealed that immune-related gene signatures are associated with survival outcomes and therapeutic efficacy, particularly in the context of immunotherapy (5). However, due to intertumoral and intratumoral heterogeneity, these prognostic genes often exhibit inconsistent performance across BC subtypes, underscoring the need for integrative analytical strategies that combine transcriptomic profiling with functional and cellular-level validation (6, 7).

Vinorelbine, a semi-synthetic vinca alkaloid, is widely used in the treatment of advanced and metastatic BC. Its primary antitumor mechanism involves binding to tubulin, thereby inhibiting microtubule polymerization and inducing mitotic arrest at the M phase, ultimately suppressing tumor cell proliferation (8, 9). Clinically, vinorelbine has demonstrated favorable efficacy and tolerability, particularly in elderly patients or those with poor performance status, for whom monotherapy can provide disease control with manageable toxicity (9). Beyond its cytotoxic effects, emerging evidence suggests that vinorelbine may exert immunomodulatory functions by influencing immune cell behavior and cytokine production within the TME, potentially contributing to enhanced antitumor immunity (10, 11). These findings display that Vino-RGs may play important roles not only in tumor cell proliferation but also in shaping the immune landscape of BC, warranting further systematic investigation (12).

In oncology research, single-cell RNA sequencing (scRNA-seq) has been increasingly adopted to refine prognostic gene discovery, elucidate immune cell dynamics, and improve the prediction of therapeutic responses (13). Integrative analyses combining scRNA-seq with bulk transcriptomic data have further enhanced the accuracy of prognostic modeling and provided novel insights into tumor–immune interactions in BC (14, 15).

This study systematically analyzed prognostically relevant genes associated with vinorelbine based on batch transcriptomic datasets, single-cell transcriptomic analysis, and experimental validation. A risk prediction model was constructed using risk score factors (RSFs) for prognostic forecasting. Subsequently, we conducted follow-up studies including functional enrichment analysis and immune microenvironment profiling. Furthermore, scRNA-seq was employed to explore cellular heterogeneity and identify key immune cell populations associated with prognostic genes. Finally, RT-qPCR validated transcriptional alterations in tumor tissues, aiming to provide novel research directions and clinical insights for breast cancer diagnosis and treatment.

## 2 Methods and materials

### 2.1 Data collection

To investigate the prognosis of BC, a training cohort was obtained from TCGA database on December 16th, 2024. This training cohort comprised RNA-sequencing data, somatic mutation data, survival information, and clinical feature data of breast tissue samples from 1,091 BC patients and 112 normal specimens. Meanwhile, the GSE42568 dataset, which comprised the transcriptome data and survival information of 104 BC patients, was recruited from GEO database. In addition, the GSE245601 dataset, containing ten breast tissue samples and two normal samples, was fully mined from the GEO database as a scRNA-seq set. It was used for single-cell analysis, which could provide a more in-depth understanding of the cellular heterogeneity in BC. To validate the spatial distribution characteristics of key signals, the spatial transcriptomics dataset GSE210616 was utilized (16), comprising tumor samples from 22 breast cancer patients (15 African-American and 7 Caucasian). Except for patient 19, two tissue sections were collected per patient, yielding 43 samples in total. Among these, 28 sections corresponded to 14 primary triple-negative breast cancer (TNBC) tumors and underwent Visium sequencing.

Furthermore, 48 candidate Vino-RGs were retrieved from the DGIdb and CTD databases (**Supplementary Table 1**). These genes represent potential associations with vinorelbine treatment efficacy in breast cancer based on existing literature and database annotations, though direct evidence of their role in vinorelbine response remains to be validated.

### 2.2 Determination and functional analysis of candidate genes

In the training cohort, the DEGs (BC vs normal) (|log_2_FC| > 0.5 and p.adjust < 0.05) were detected via the DESeq2 package (v 1.38.0) (17). Subsequently, all DEGs were displayed in a volcano plot, and the top ten up-regulated DEGs with the highest log_2_FC and top ten down-regulated DEGs with the lowest log_2_FC were labeled applying the ggplot2 package (v 3.3.6) (18). Meanwhile, these 20 DEGs marked in the volcano plot were also displayed in a heatmap applying the ComplexHeatmap package (v 2.14.0) (19).

Subsequently, to ascertain the genes related to vinorelbine that played significant roles in BC development, the overlapping genes between DEGs and Vino-RGs were acquired and designated as CGs employing the ggvenn package (v 0.1.10) (20). Subsequently, GO and KEGG enrichment analyses (p < 0.05) were carried out via the clusterProfiler package (v4.7.1.3) (21). The top five significant GO terms in each catalog and the top five KEGG pathways with the lowest p were respectively shown applying the GOplot package (v 1.0.2) (22).

Relied on the STRING, the interactions among the proteins coded for CGs were detected (confidence score = 0.4). Then, the protein-protein interaction (PPI) network was depicted applying the Cytoscape package (v 0.1.10) (23) after removing discrete genes.

### 2.3 Identification of prognostic genes

Based on training data, univariate Cox regression analysis was executed using CGs, with ps < 0.2. Additionally, the proportional hazards (PH) assumption test was executed, where a p > 0.05 indicated that the samples proceeded the PH assumption test. These analyses were conducted to ascertain the genes linked to the prognosis of BC patients applying the survival package (v 3.7.0) (24). The results were presented in a forest plot employing the forestplot package (v 3.1.3) (25). Genes with an HR above 1 were designed as risk factors for BC, while those with an HR below 1 were defined as protective factors. Subsequently, the LASSO analysis was employed via the glmnet package (v 4.1.8) (26). In this process, ten-fold cross-validation was employed to calculate the lambda value, and the prognostic genes were mined with the lambda reaching the minimum value.

### 2.4 Establishment and validation of risk model

Relied on the prognostic genes, the randomForestSRc package (v 3.2.3) (27) was employed to build a RSF model in TCGA-BRCA dataset. This model was deemed as risk model, and the risk score of each patient was acquired.

Thereafter, to evaluate the performance of the risk model, 1,091 BC patients from the TCGA-BRCA dataset were stratified into HRG and low-risk (LRG) groups relied on the optimum cutoff of the risk score. Risk curves were then generated to clearly visualize the spread situation of risk scores, and scatter plots were clearly created to illustrate the survival status of BC patients in both HRG and LRG. After, K-M curves were depicted applying the “survminer” package (v 0.4.9) to assess survival diversity between the HRG and LRG (p < 0.05, log-rank test). Additionally, survival rate ROC curves were built applying the “timeROC” package (v 0.4), and the AUC was clearly calculated to measure the projected accuracy of the risk model. To enhance clinical robustness and mitigate cohort size constraints, we adopted nested cross-validation, broadened performance metrics, and benchmarked models against standard clinical predictors (28). Specifically, nested 5-fold cross-validation was employed: outer folds evaluated generalization performance on held-out samples, and inner folds tuned hyperparameters. A random survival forest model was built via the randomForestSRC package (v 3.2.3). Model performance was assessed via Harrell’s concordance index (C-index), time-dependent ROC curves (plotted with the timeROC package v 0.4), Brier scores, and integrated Brier scores (IBS, calculated via the riskRegression package). Calibration curves were generated using ggplot2 package.

To fully validate the robustness of the risk model, the same analyses were executed on the GSE42568 dataset (comprising 104 samples with complete survival information), following the identical methods and steps.

### 2.5 Establishment and appraisal of a nomogram

To probe the correlations of clinical features with the risk score, 1,091 BC samples in TCGA-BRCA cohort were first divided into different subgroups relied on age, gender, tumor (T) stage, metastasis (M) stage, node (N) stage, and clinical stage. Then, the risk score differences between different subgroups were detected by Wilcoxon test (p < 0.05). Subsequently, the univariate Cox regression analysis (HR ≠ 1 and p < 0.05) and the PH assumption test (p > 0.05) were used again to check the factors linked to the prognosis of BC from the risk score and six clinical features analyzed applying the survival package. After that, the multivariate Cox regression analysis (HR ≠ 1 and p < 0.05) was performed using the 1,091 BC samples and the survival package. Furthermore, a nomogram was built via the rms package (v6.5.0) (29) to project 1-, 2-, and 3-years mortality rates in breast cancer patients. ROC curves and calibration curves were yielded to evaluate the projected exactness of the nomogram applying the timeROC package and the rms package. In the ROC curves, an AUC greater than 0.6 meant good predictive accuracy for this model.

### 2.6 Function enrichment analyses

To fully investigate the pathways that functioned during BC progressed, the 1,091 samples in the TCGA-BRCA dataset were employed again to perform the GSEA. First, background gene cohort was acquired from MSigDB. Subsequently, the DESeq2 was applied to calculate the log_2_FC for each gene between the two risk groups, and the genes were ranked according to the log_2_FC in ascending. Furthermore, the GSEA was executed employing the clusterProfiler package, with the criterion of p.adjust < 0.05.

In addition, relied on the training data, the GSVA package (v 1.50.0) (30) was deployed to measure the GSVA score employing the background gene cohort “h.all.v2024.1.Hs.symbols.gmt” from the MSigDB database. Then, the limma package (v 3.58.1) (17) was implemented to analyze the diversity in the score between two risk cohorts with the criterion of |t| > 2, p < 0.05.

### 2.7 Immune microenvironment analysis

To provide more references for BC treatment and prognosis, the relative infiltration abundances of 28 immune cells (31) based on HRG and LRG in the training data were detected by the ssGSEA algorithm (v 0.99.8) (32). The immune cells with notable abundance differences between the two risk cohorts were ascertained as differential immune cells employing the Wilcoxon test (p < 0.05). The correlation between differential immune cells and prognostic genes, as well as risk score, was ascertained by the Spearman correlation analysis (|correlation (cor)| > 0.3 and p < 0.05) employing the psych package (v 2.4.3) (33).

Beyond the analysis of immune cell infiltration, the characteristics of the TME were also investigated. Three TME scores, including the immune, ESTIMATE, and stromal score, were first measured by the ESTIMATE algorithm (v 1.0.13) (34) based on the training cohort. Subsequently, the discrepancies in the three scores between the HRG and LRG were detected (p < 0.05).

### 2.8 Tumor immune dysfunction and exclusion analysis

The TIDE scores of samples in the two risk groups of the training cohort were calculated applying the TIDE database. The difference in TIDE scores between the two risk groups was analyzed applying the Wilcoxon test (p < 0.05). The correlation between risk score and TIDE score was also identified using the Spearman correlation analysis (|cor| > 0.3, p < 0.05).

In addition to TIDE analysis, the IPS was also investigated to further evaluate the immunotherapy effect in BC. Subsequently, the IPS, including ips_ctla4_pos_pd1_pos, ips_ctla4_pos_pd1_neg, and ips_ctla4_neg_pd1_pos, of samples in the two risk cohorts were downloaded from TCIA to analyze the treatment effect of CTLA4 and PD1 in BC. The difference in IPS between the two risk cohorts was analyzed via the Wilcoxon test (p < 0.05).

### 2.9 Somatic mutation analysis

To explore the potential mechanism of BC development, the somatic mutation data of the 1,091 BC samples in the training cohort were downloaded via the TCGA database. The Maftools package (v 2.18.0) (35) was adopted to evaluate the TMB score and assess the mutation profiles of the two risk groups in the training cohort. The top 20 genes with the highest mutation repetition in the two risk cohorts were ascertained. The difference in TMB score between the two risk cohorts was analyzed applying the Wilcoxon rank-sum test (p < 0.05). Thereafter, the correlation between TMB and risk score was also ascertained (p < 0.05).

### 2.10 Drug sensitivity analysis

In addition to exploring the somatic mutation patterns, understanding drug sensitivity relied on the prognostic genes was also crucial for BC research. The IC_50_ could measure the ability of drugs to induce apoptosis. Based on the expression situation of the prognostic genes among the risk groups in the training cohort, the IC_50_ value of 138 drugs for each specimens was computed via the pRRophetic package (v 0.5) (36). The correlations between drugs and risk score were fully analyzed. The IC_50_ differences of drugs correlated with the risk score between the two risk cohorts were also detected via the Wilcoxon test (p < 0.05), and displayed by the ggplot2 package. To further explore the potential association between candidate genes and vinorelbine sensitivity, we analyzed the correlation between the expression levels of the eight prognostic genes and the predicted half-maximal inhibitory concentration (IC_50_) of vinorelbine using the pRRophetic package (v 0.5).

### 2.11 Single-cell analysis

Using the Seurat package (v 5.0.1) (37), we performed single - cell analysis. We selected eligible cells and genes from GSE245601 relied on the following criteria: i) Genes detected in less than three cells were removed; ii) Cells with fewer than 200 or more than 6,000 genes were fully removed; iii) Cells with total count less than 500 or more than 10,000 were removed; iv) Cells with above 10% of mitochondrial genes were also eliminated. After the eligible cells and genes were selected, the LogNormalize function was adopted to normalize the data. Then, 2,000 genes with the highest expression variations were collected for further analyses by the FindVariableFeatures function. These genes were visualized in a scatter plot. The top ten genes with the highest variations were denoted on the plot. Next, data normalization was accomplished again using the ScaleData function. The PCA was performed to identify the optimal PCs for cell clustering using the runPCA, JackStrawPlot, and JackStraw functions. Based on the optimal PCs, cell clustering (resolution = 0.2) was completed using the FindClusters and FindNeighbors functions. The clustering results were displayed applying the UMAP method.

After that, cell annotation was accomplished basis of the expression of markers from the Cellmarker2.0 database using the FindAllMarkers function. The results were shown using the Dotplot function. After cell annotation, differential cells were identified by comparing the abundance of annotated cells between BC and normal samples applying the Wilcoxon test (p < 0.05). Crucial cells were further ascertained by selecting the differential cells with the highest expression levels of prognostic genes.

### 2.12 Cell-cell communication analysis

For the spatial transcriptomics dataset GSE210616, this study performed preprocessing and integration analyses using Scanpy (v1.11.4) (38). Raw 10x Visium data (including expression matrices, spatial coordinates, and low-resolution tissue images) from each sample were first loaded via sc.read_visium(), and then merged into a unified object using sc.concat(). Subsequently, standard processing steps were applied: filtering lowly expressed genes (retaining genes detected in at least 3 spots), normalizing total transcript counts to 10,000 (sc.pp.normalize_total), log-transforming expression values (sc.pp.log1p), identifying highly variable genes (sc.pp.highly_variable_genes) and retaining this subset for downstream analysis, and scaling data to constrain values within the range of [-10, 10] (sc.pp.scale). Based on the processed data, principal component analysis (PCA) was performed to extract the top 30 principal components. To eliminate batch effects across samples, harmony-py was employed to integrate the data using the “sample” label as a covariate. Finally, a neighborhood graph was constructed based on the integrated PCA results, followed by UMAP dimensionality reduction and Leiden clustering to obtain spot-level cell cluster labels. This study supplemented 42 TNBC patients’ spatial transcriptomic data (GSE210616, totaling 55,670 spots) and inferred cell abundance using cell2location (39). Analyses were strictly stratified by patient: Spearman correlations between TGFB1/macrophage signatures and cell abundances were first calculated within each patient sample, then averaged across 42 cases. All data were normalized via Seurat v4.3’s SCTransform and batch-corrected using Harmony to eliminate batch effects and tissue dissociation biases[PMID: 40824857]. Cell type annotations were based on published single-cell reference matrices to ensure consistency.

### 2.13 Crosstalk analysis between TGFB1 and other prognostic genes.

This study systematically evaluated the expression correlation between TGFB1 and seven other prognostic genes (XRCC1, BRCA1, IL7, TUBB2B, TUBA1B, TUBA1C, PTGS2), as well as the associations of TGFB1 with immune infiltration features, immune checkpoint molecules, and TIDE-related immunotherapy indicators (40). Spearman correlation was used, and ps were corrected by the Benjamini-Hochberg method. Significance was defined as FDR < 0.05, and network edge filtering criteria were FDR < 0.05 and |Spearman r| ≥ 0.30. All analyses were performed based on TCGA-BRCA cohort data.

### 2.14 Cell-cell communication analysis

The communication types among tagged cells were sensed applying the CellChat package (v 1.6.1) (41). The interactions of ligand-receptor among distinct cells was observed and illustrated in a bubble plot by the ggplot2 package.

### 2.15 Pseudotime analysis

In this study, the differentiation trajectories of crucial cells were probed using the monocle package (v 2.26.0) (42). First, cell clustering of crucial cells was performed with a resolution of 0.5. The UMAP method was applied to visualize the results. Subsequently, the differentiation trajectory of each crucial cell was ascertained, and expression trends of the prognostic genes in the whole differentiation state were also detected. Plot key cell distribution across patients with ggplot2 package stacked bars.

### 2.16 Construction of murine tumor models and treatment regimen

Murine BC (4T1) cells and Lewis lung carcinoma (LLC1) cells were sourced from the American Type Culture Collection (ATCC) and cultured basis for suppliers’ directions. LLC1 cells were maintained in DMEM medium (Vivacell, C3113-0500), while 4T1 cells were cultured in dedicated 4T1 medium (Zhongqiao Xinzhou, ZM0201), both supplemented with 10% fetal bovine serum (FBS; Vivacell, C04001-500) in a humidified incubator containing 5% CO_2_ at 37 °C.

For subcutaneous tumor establishment, cells were captured, washed twice with serum-free medium, and resuspended at a density of 1 × 10^7^ cells/mL. A total volume of 100 μL cell suspension was administered subcutaneously into the right lower flank of mice. 4T1 cells were inoculated into BALB/c mice, and LLC1 cells were inoculated into C57BL/6 mice.

On day 6 after tumor inoculation, mice were randomized into different treatment groups and weighed. Tumor volume and body weight were monitored throughout the experimental period. Vinorelbine tartrate (5 mg/kg; Chenguang Biotech, 125317-39-7) was administered intraperitoneally on days 7, 9, and 11 to the vinorelbine and combination groups, while control and anti-PD-1 groups received no vinorelbine. Anti-PD-1 antibody (100 μg per mouse; BioXCell, BE0146) was administered intravenously on days 13, 15, and 17 to the anti-PD-1 and combination groups, while control and vinorelbine groups received no antibody.

Mice were observed for survival after completion of treatment. Tumor volumes exceeding 2000 mm³ were defined as humane endpoints. All animal procedures were authorized by the Animal Ethics Committee of Kunming Medical University (Approval No. kmmu20221238) and executed in conformity with institutional protocols.

### 2.17 Isolation and differentiation of bone marrow–derived macrophages

BMDMs were generated from 8–10-week-old BALB/c mice. Femurs and tibias were aseptically isolated, and surrounding muscle tissues were removed. Bones were sterilized in 75% ethanol for 5 min and washed twice with sterile phosphate-buffered saline (PBS). Bone marrow cells were flushed out using RPMI-1640 medium and passed through a 70-μm cell strainer. After centrifugation, red blood cells were lysed via red blood cell lysis buffer, followed by washing with RPMI-1640 medium. Cells were farmed in RPMI-1640 medium supplemented with 10% FBS and recombinant mouse macrophage colony-stimulating factor (M-CSF, 20 ng/mL; Sino Biological, 51112-MNAH). On day 6, cells were cultured for an additional 24 h in medium containing either M-CSF alone or M-CSF plus interleukin-4 (IL-4, 20 ng/mL; Sino Biological, 51084-MNAE) to induce M2-like polarization. On day 7, differentiated macrophages were harvested for subsequent experiments. Macrophage differentiation and polarization were confirmed by morphological assessment and flow cytometric analysis.

### 2.18 Flow cytometric analysis

Differentiated BMDMs were reacted with vinorelbine at various concentrations (0.01–100 nM) for 24 h. Cells were gathered by gentle scraping, washed twice with PBS, and resuspended for staining. Cells were incubated with Fc Block (1 μL per sample; BD Pharmingen, 553141) for 10 min on ice, followed by staining with fluorochrome-conjugated antibodies: FITC anti-mouse CD11b (BD Pharmingen, 557396), PE anti-mouse F4/80 (BD Pharmingen, 565410), PE-Cy7 anti-mouse CD86 (BD Pharmingen, 560582), and Alexa Fluor 647 anti-mouse CD206 (BD Pharmingen, 565250). After incubation for 30 min on ice, cells were washed three times with PBS and analyzed using a NovoCyte flow cytometer.

### 2.19 Enzyme-linked immunosorbent assay

BMDMs were treated with vinorelbine at the revealed concentrations for 24 h. Culture supernatants were collected and concentrated by 10 kDa molecular weight cutoff centrifugal filters (Millipore, UFC801096) by centrifugation at 5000 g for 10 min per cycle until a final volume of approximately 1 mL was gained. The concentrations of cytokines in the supernatants were appraised by commercial ELISA kits according to the manufacturers’ instructions, including Mouse TGF-β1 ELISA Kit (NeoBioscience, EMC107b.96), Mouse IL-10 ELISA Kit (NeoBioscience, EMC005.96), Mouse TNF-α ELISA Kit (NeoBioscience, EMC102a.96), and Mouse IL-6 ELISA Kit (NeoBioscience, EMC004.96).

### 2.20 RT-qPCR

We executed PCR testing on 3 BC, 3 BC-VLB (treated with vinorelbine), 3 BC-PD (treated with PD-1 inhibitor), and 3 BC-VLB+PD specimens (treated with both vinorelbine and PD-1 inhibitor) obtained from the experimental animals in Section 2.16. First, total RNA was isolated from specimens via TRIzol reagent (Ambion, USA). Subsequently, cDNA was synthesized via the HP All-in-one qRT Master Mix II RT203-Ver. 1 Kit (Kunming Yungen Biotechnology Co., Ltd.). Primer sequences are listed in **Supplementary Table 2**, qPCR was executed on the CFX96 Connect Real-Time Quantitative PCR System (Bio-Rad, USA). Relative mRNA expression levels were measured by the 2^-ΔΔCT^ method. Finally, results were deployed to Excel and statistically analyzed and depicted by GraphPad Prism 10.

### 2.21 Statistical analysis

All analyses were executed with the R package (v 4.2.2). The Wilcoxon signed-rank test was adopted to measure intergroup differences (p < 0.05). Experimental data were analyzed by GraphPad Prism (version 10.0). Comparisons between two cohorts were carried out via the unpaired Student’s t-test, while comparisons involving three or more cohorts were executed by one-way analysis of variance (ANOVA). Tumor volume data were analyzed via Huynh-Feldt-corrected two-way ANOVA followed by Tukey’s multiple comparison test. Survival analysis deployed K-M survival curves contrasted via log-rank test (Mantel-Cox). For RT-qPCR analysis, statistical comparisons utilized Student’s t-test. *P<0.05.

## 3 Results

### 3.1 Identification of eight prognostic genes for BC

**Figure 1:**
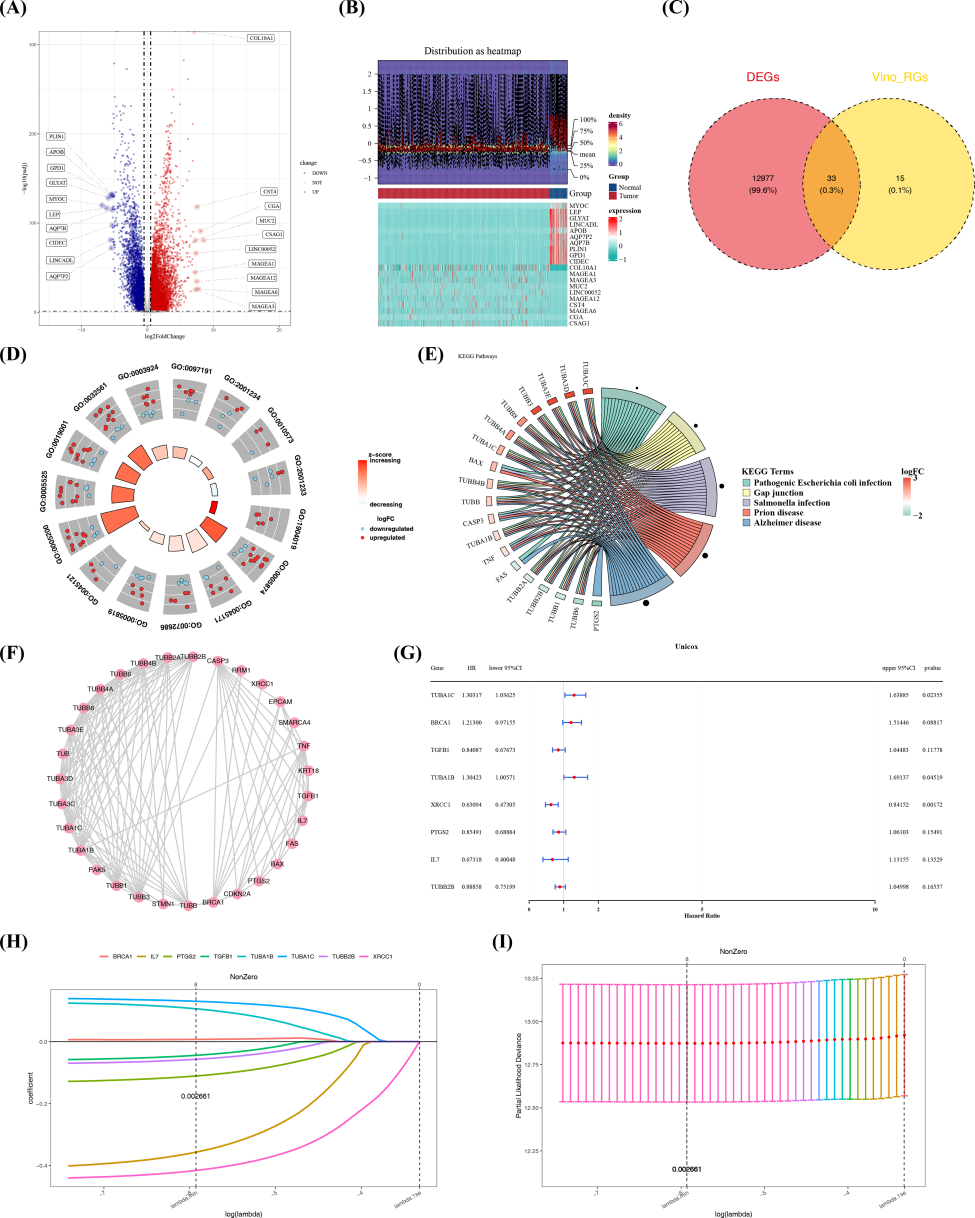
Identification and functional characterization of candidate vinorelbine-related genes in breast cancer. **(A)** Volcano plot of differentially expressed genes between breast cancer and normal tissues. **(B)** Heatmap of differentially expressed genes. **(C)** Intersection of differentially expressed genes and vinorelbine-related genes to identify candidate genes. **(D)** Gene Ontology enrichment analysis of candidate genes. **(E)** Kyoto Encyclopedia of Genes and Genomes pathway enrichment analysis of candidate genes. **(F)** Protein–protein interaction network of candidate genes. **(G)** Univariate Cox regression analysis identifying prognosis-related candidate genes. **(H)** Least absolute shrinkage and selection operator coefficient profiles of the prognosis-related genes. **(I)** Cross-validation for optimal lambda selection in the least absolute shrinkage and selection operator model.

After the differential expression analysis, 13,010 DEGs were detected between BC and normal specimens (**Figures 1A,B**; **Supplementary Table 3**). Among these genes, 8,414 DEGs showed higher expression levels and 4,596 DEGs showed lower expression levels in BC samples than in normal samples. Subsequently, 33 CGs were ascertained by overlapping the 13,010 DEGs and 48 Vino-RGs, which provided a solid foundation for investigating the roles of Vino-RGs in BC (**Figure 1C**; **Supplementary Table 4**).

For these 33 CGs, 818 GO terms, including 732 BP, 37 CC, and 49 MF, as well as 66 KEGG pathways were enriched (**Supplementary Tables 5**, **6**). These results indicated that the CGs might affect BC development through functions and pathways such as the “extrinsic apoptotic signaling pathway”(BP), “microtubule” (CC), “guanyl nucleotide binding” (MF), as well as “gap junction” and “salmonella infection” in KEGG (**Figures 1D, E**).

A PPI network was then built, which revealed that the proteins encoded by 31 out of the 33 CGs exhibited various interactions with other proteins in the network (**Figure 1F**). Among them, TUBB2B interacted with multiple proteins, such as TUBB1 and TUBA3E. Interestingly, the PPI network suggested that members of the TUBB gene family might is essential for development of BC. Furthermore, only eight CGs, including TUBA1C, BRCA1, TGFB1, TUBA1B, XRCC1, PTGS2, IL7, and TUBB2B, were verified to be correlated with the prognosis of BC (**Figure 1G**; **Table 1**). Among them, TUBA1C and TUBA1B were determined as risk factors for BC (HR > 1 and p < 0.2). Afterwards, all eight genes were identified as prognostic genes for BC patients when the lambda reached the minimum value (0.002661) (**Figures 1H, I**).

**Table 1:** Proportional hazards assumption test results for the eight prognostic vinorelbine-related genes in breast cancer.

	Gene	chisq	df	p
2	TUBA1C	2.113264273	1	0.146027535
17	BRCA1	1.018124956	1	0.312964188
19	TGFB1	0.000999212	1	0.974782822
20	TUBA1B	1.10848324	1	0.292411896
21	XRCC1	0.071531215	1	0.789120241
26	PTGS2	0.734181661	1	0.391531098
30	IL7	2.137048448	1	0.143778286
32	TUBB2B	2.723851501	1	0.09885921

### 3.2 The RSF model for the prognosis of BC patients

**Figure 2:**
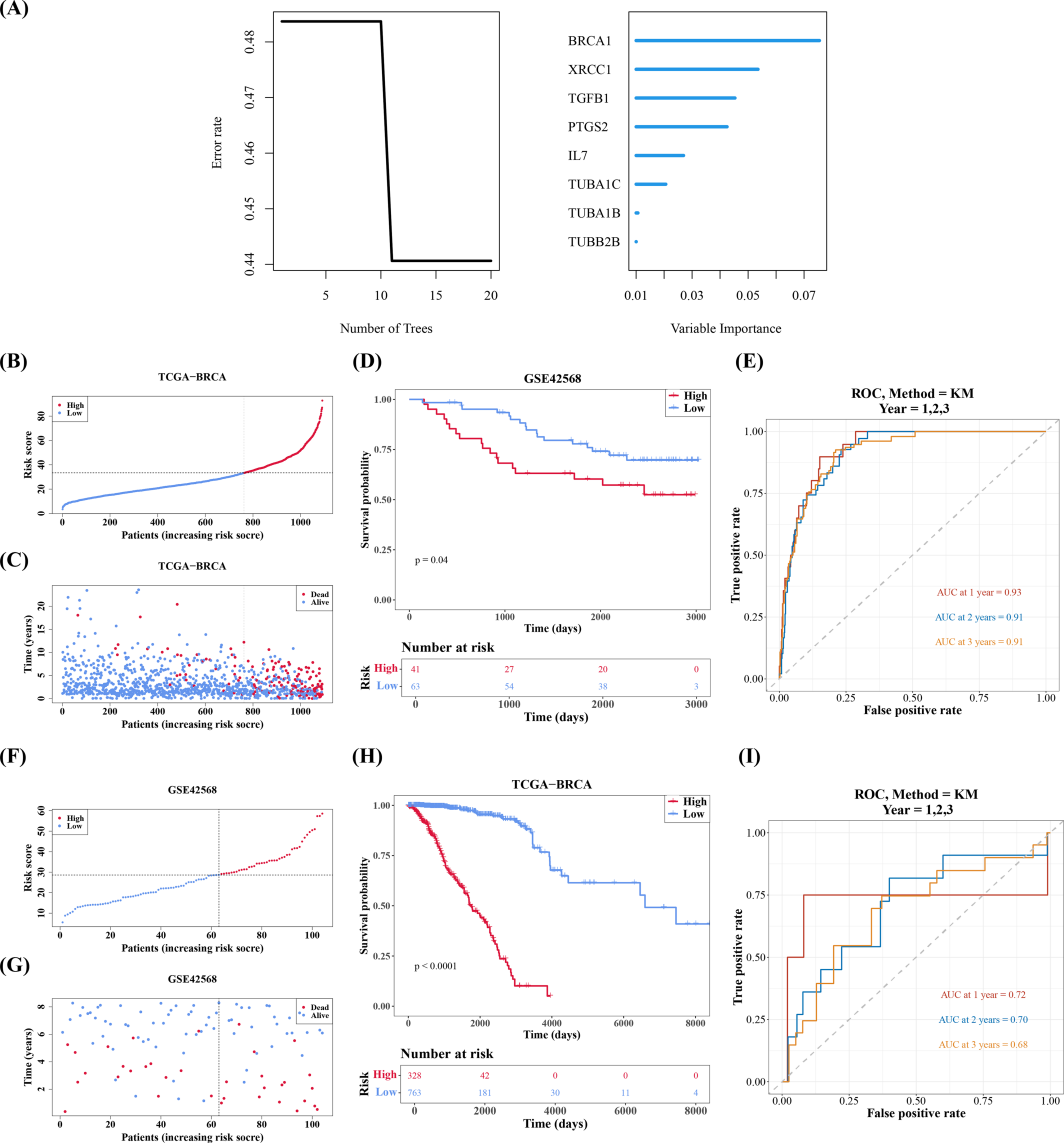
Construction and validation of the random survival forest prognostic model for breast cancer. **(A)** Variable importance of the eight prognostic genes in the random survival forest model. **(B)** Distribution of risk scores and cutoff value in the training cohort. **(C)** Survival status and follow-up time of patients in the training cohort. **(D)** Kaplan–Meier survival curves for the high- and low-risk groups in the training cohort. **(E)** Time-dependent receiver operating characteristic curves of the model in the training cohort. **(F)** Distribution of risk scores and cutoff value in the validation cohort. **(G)** Survival status and follow-up time of patients in the validation cohort. **(H)** Kaplan–Meier survival curves for the high- and low-risk groups in the validation cohort. **(I)** Time-dependent receiver operating characteristic curves of the model in the validation cohort.

In the RSF model, BRCA1 had the greatest importance among eight prognostic genes, suggesting it was more important for the accuracy ability of the model (**Figure 2A**). Moreover, in the model, the 1,091 BC samples in the training cohort were divided into the HRG (328 samples) and the LRG (763 samples) referring to the optimal cut-off value of 33.32323 (**Figure 2B**). Between the two risk groups, the HRG had more deceased samples with shorter survival times than those in the LRG (**Figure 2C**). After that, the K-M survival curves revealed that the survival probability of samples in the LRG was notably higher than that in the HRG (p < 0.0001) (**Figure 2D**). The AUC values were respectively 0.93, 0.91, and 0.91, which suggested that the RSF model was highly accurate for the prognosis of BC (**Figure 2E**).

Additionally, the 104 BC samples in the validation cohort were also divided into the HRG (41 samples) and the LRG (63 samples) referring to the optimal cut-off value of 28.66738 (**Figure 2F**). Between these two risk groups in the validation cohort, a consistent and significant difference in survival probability similar to that in the training cohort was also detected (p = 0.04) (**Figures 2G, H**). The AUC values were respectively 0.72, 0.70, and 0.68 (**Figure 2I**). These findings further validated the predictive reliability of the risk model for the prognosis of BC patients.

**Figure 3:**
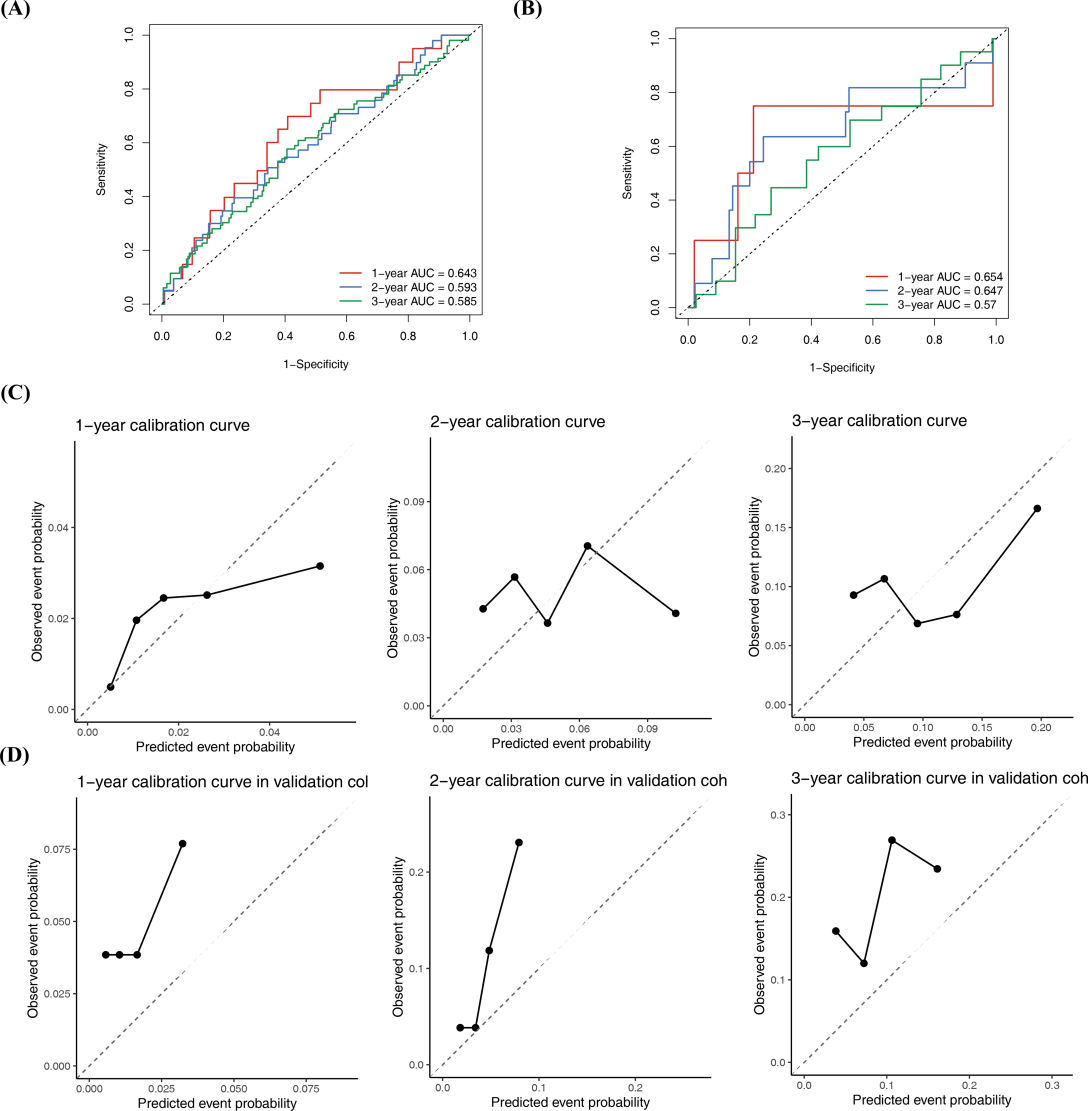
Performance and validation of the predictive model for the prognosis of breast cancer patients. **(A, B)** Time-dependent ROC curves for the training set (left) and validation set (right). The 1-, 2-, and 3-year AUCs were 0.643, 0.593, and 0.585 in the training set, and 0.654, 0.647, and 0.570 in the validation set, confirming acceptable discriminatory ability. **(C, D)** Calibration curves demonstrating consistent trends between predicted and observed probabilities, with excellent calibration performance particularly evident in the 1-year model.

Nested cross-validation demonstrated stable performance (**Table 2**). For the Nested RSF model, Brier scores at 1/2/3 years were 0.0206/0.0482/0.0924, with IBS of 0.0000/0.0103/0.0229, indicating low prediction error. Time-dependent ROC curves (**Figures 3A,B**) showed 1/2/3-year AUCs of 0.643/0.593/0.585 (training set) and 0.654/0.647/0.570 (validation set), confirming acceptable discrimination. Calibration curves (**Figures 3C,D**) demonstrated consistent trends between predicted and observed probabilities, especially for the 1-year model. The C-index of the nested cross-validation model was 0.54, while that of the external validation cohort was 0.55, indicating good performance. External validation on GSE42568 yielded 1/2/3-year AUCs of 0.61/0.58/0.53, Brier scores of 0.046/0.100/0.179 (**Table 3**), supporting generalizability despite cohort size.

**Table 2:** Predictive performance of the prognostic models evaluated by nested cross-validation.

Model	Times	Brier	se	Lower	Upper	IBS
Null model	365	0.0204632306027401	0.00433011249337365	0.0119763660667208	0.0289500951387594	0
Null model	730	0.0470079271172938	0.00672221332456187	0.0338326311047573	0.0601832231298304	0.0102316153013701
Null model	1095	0.0913489672808767	0.00940517989488896	0.0729151534187741	0.109782781142979	0.022490385906678
Nested RSF	365	0.0206253823236537	0.00428800003784077	0.0122210566837794	0.029029707963528	0
Nested RSF	730	0.0481943050962157	0.00671364287518625	0.0350358068557867	0.0613528033366447	0.0103126911618268
Nested RSF	1095	0.0923641875119655	0.00924097208847772	0.0742522150364093	0.110476159987522	0.0229398958066231

**Table 3:** Brier scores of the prognostic model in the external validation cohort (GSE42568).

Time	Days	pred_col	n_total	n_used	n_excluded_due_to_censoring	Brier
1-year	365	pred_event_1y	104	104	0	0.046417681127031
2-year	730	pred_event_2y	104	101	3	0.0995970454753609
3-year	1095	pred_event_3y	104	98	6	0.179331800775808

### 3.3 The nomogram for the prognosis of BC patients

**Figure 4:**
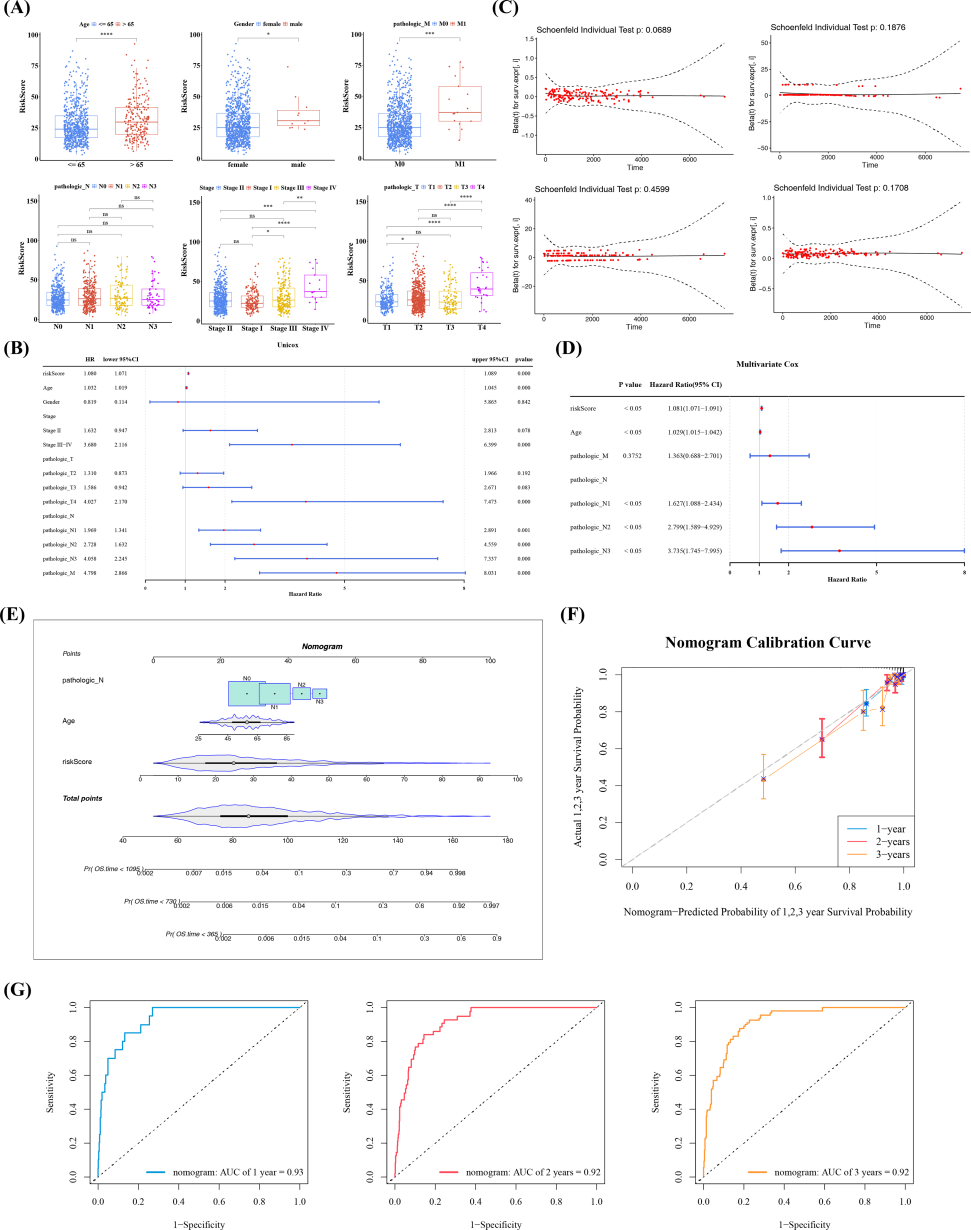
Construction and evaluation of the prognostic nomogram for breast cancer. **(A)** Differences in risk score among clinical subgroups. **(B, C)** Cox regression analyses of clinicopathological variables and risk score associated with overall survival. **(D)** Independent prognostic factors included in the nomogram. **(E)** Nomogram for predicting 1-, 2-, and 3-year overall survival. **(F)** Calibration curves of the nomogram. **(G)** Time-dependent receiver operating characteristic curves of the nomogram.

After detecting the diversity in risk score among clinical features, patients >65 years had notably higher risk scores than those ≤ 65 years old (p < 0.0001) (**Figure 4A**). Male BC patients had notably higher risk scores than female BC patients (p < 0.05). Moreover, as BC developed, the risk score generally showed an upward trend. Subsequent analyses verified that the risk score, age, M, and N stage were related to the prognosis of BC (**Figures 4B, C**). Further analyses selected the risk score, age, and N stage as the independent prognostic factors (**Figure 4D**). The nomogram of these three independent prognostic factors showed that these factors all had high predictive performance for the death rate of BC patients, especially the risk score (**Figure 4E**). Subsequently, the slopes of all calibration curves were close to 1 (**Figure 4F**), and the AUC of ROC curves were respectively 0.93 (1 year), 0.92 (2 years), and 0.92 (3 years) (**Figure 4G**). These findings verified that the nomogram had good predictive accuracy for the death rate of BC patients, supporting its potential clinical utility in risk-stratified management of breast cancer.

### 3.4 Enrichment analysis for BC

**Figure 5:**
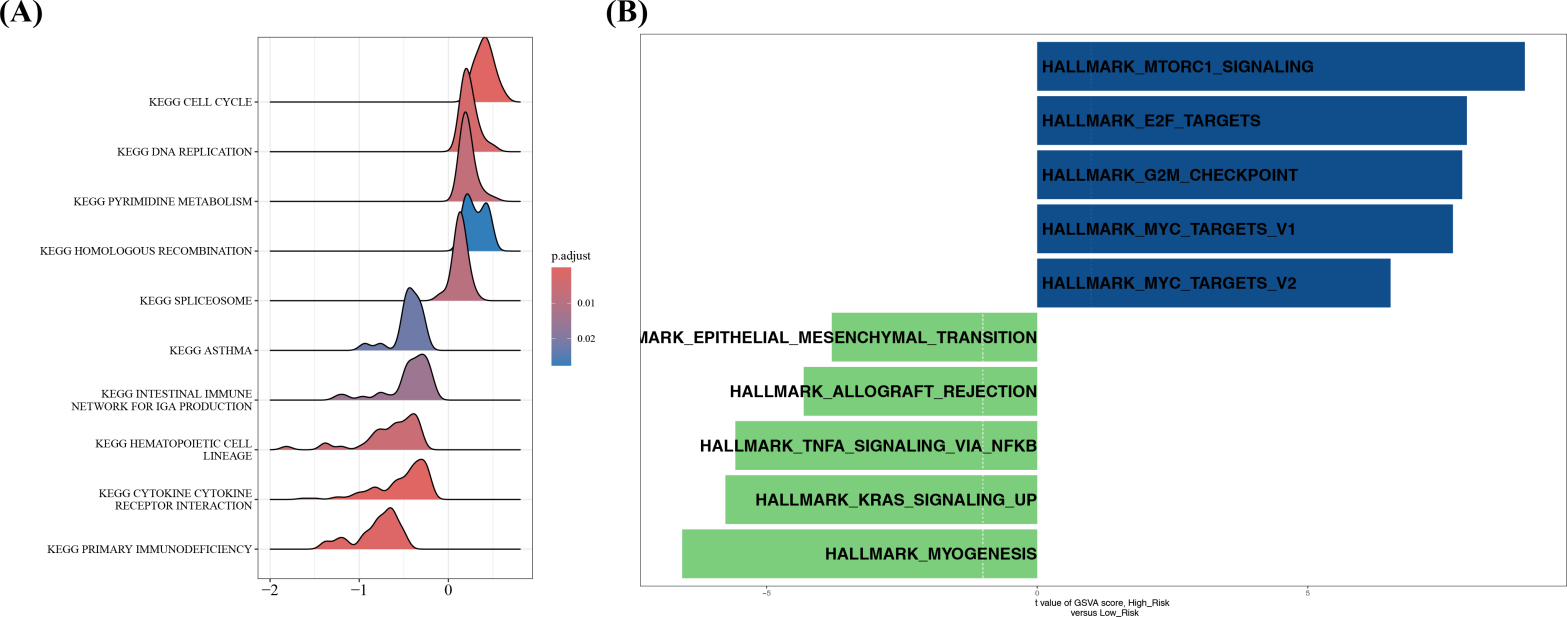
Functional pathway alterations associated with risk stratification in breast cancer. **(A)** Gene Set Enrichment Analysis comparing the high- and low-risk groups. **(B)** Gene Set Variation Analysis showing activated and inhibited hallmark pathways between the two risk groups.

As BC developed, a total of 29 pathways were enriched by GSEA (**Supplementary Table 7**). This enrichment analysis revealed that multiple pathways were implicated in in BC development, such as “cell cycle”, “pyrimidine metabolism”, and “steroid biosynthesis” (**Figure 5A**).

In addition to the GSVA results, 42 pathways were enriched for BC (**Supplementary Table 8**). Among these, 19 pathways were activated as BC progressed, such as “mTORC1 signaling”, “unfolded protein response”, and “E2F targets”. Meanwhile, 23 pathways were inhibited, such as “myogenesis”, “down-regulated KRAS signaling”, and “up-regulated KRAS signaling” (**Figure 5B**).

### 3.5 Altered immune environment in BC development

**Figure 6:**
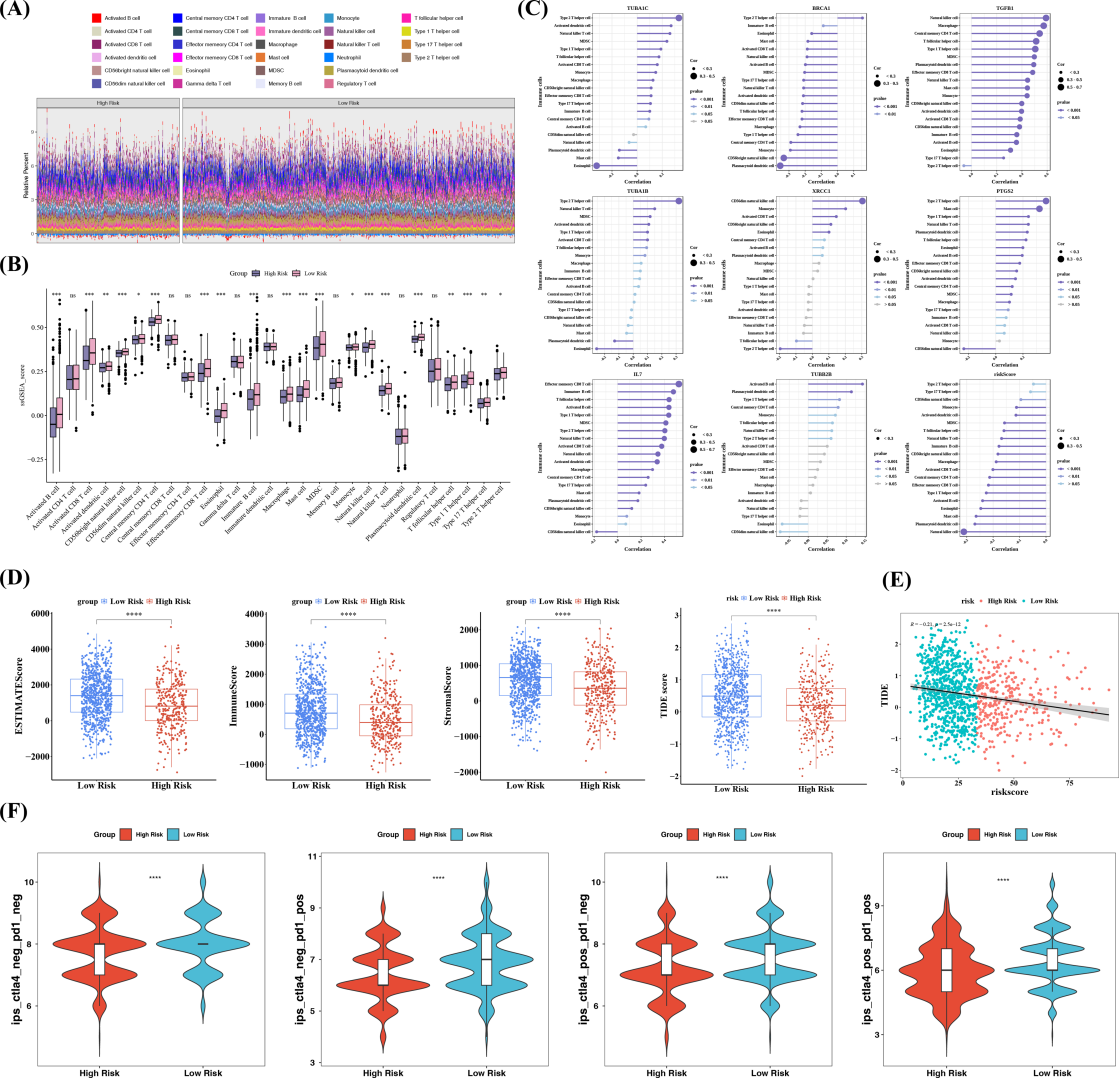
Immune landscape and immunotherapy-related characteristics of the risk groups. **(A)** Differences in infiltrating immune-cell abundance between the high- and low-risk groups. **(B)** Correlations of risk score and prognostic genes with differential immune cells. **(C)** Comparison of immune score, stromal score, and ESTIMATE score between the two risk groups. **(D)** Comparison of Tumor Immune Dysfunction and Exclusion scores between the two risk groups. **(E)** Comparison of immunophenoscores between the two risk groups. *P < 0.05, **P < 0.01, ***P < 0.001, ****P < 0.0001, and ns indicates no statistically significant difference.

Among 28 immune cells, 20 immune cells exhibited significant abundance differences (p < 0.05) (**Figures 6A,B**), such as activated CD8 T cells and macrophages. Interestingly, the risk score and BRCA1 were notably negatively correlated (cor < -0.3 and p < 0.01) with all differential immune cells except type 2 T helper cells, while TGFB1 was notably positively correlated with all differential immune cells (cor > 0.3 and p < 0.05), except type 2 T helper cells (**Figure 6C**).

In the HRG, the immune score, Estimate score, and stromal score were markedly lower than those in the LRG (p < 0.05) (**Figure 6D**). This result indicated a relatively higher levels of tumor cells in the HRG. Overall, these characteristics suggested that the tumor had stronger autonomy, immune escape ability, and metastatic potential, and predicted a poor prognosis for BC patients.

Moreover, the TIDE score in the HRG was notably lower than that in the LRG (p <0.0001). It should be noted that a lower TIDE score typically implied weaker tumor immune-escape ability (**Figure 6E**). This finding suggested that tumors in the HRG might exhibit reduced immune-escape ability compared to those in the LRG, indicating a potentially more favorable immune environment for immune-based therapies.

Furthermore, the IPS in the HRG was notably lower than that in the LRG. IPS reflects the expression levels of immune checkpoint molecules (such as CTLA4 and PD1); a lower IPS typically indicates reduced expression of these checkpoints, suggesting that HRG patients may have limited benefit from immune checkpoint blockade therapies. However, the lower TIDE score in HRG implies weaker tumor immune-escape ability, indicating a less immunosuppressive tumor microenvironment overall (**Figure 6F**). Specifically, further validation in real immunotherapy cohorts is required.

### 3.6 TMB and drug sensitivity analysis

**Figure 7:**
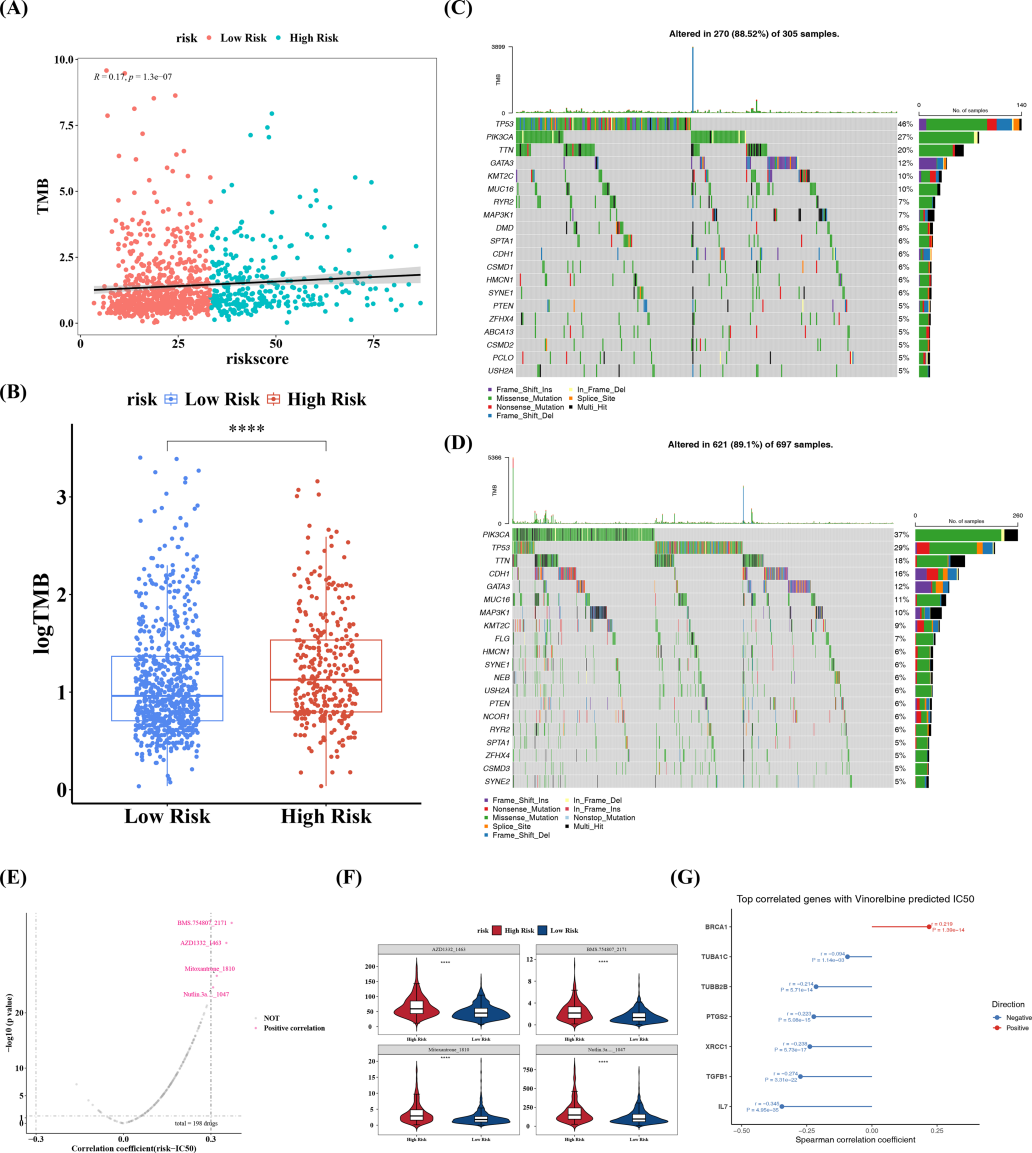
Tumor mutation burden and drug-sensitivity analyses of the risk groups. **(A)** Correlation between risk score and tumor mutation burden. **(B)** Comparison of tumor mutation burden between the high- and low-risk groups. **(C, D)** Mutation landscapes of the low-risk and high-risk groups. **(E)** Correlations between risk score and predicted half-maximal inhibitory concentrations of candidate drugs. **(F)** Comparison of predicted drug sensitivity between the high- and low-risk groups. **(G)** Correlation between 8 Prognostic Genes and Vinorelbine IC50 (Significant Associations and Direction). ****P < 0.0001.

First, only 305 BC samples in the HRG and 697 BC samples in the LRG were selected to ascertain the gene with the highest mutation frequency and assess TMB score between two risk groups, with the mutation information. A direct link between risk score and TMB score was ascertained (**Figure 7A**). Moreover, samples in the HRG had notably higher TMB scores than those in the LRG (p < 0.0001), which provided a solid evidence for the correlation between the two scores (**Figure 7B**). In the two risk groups, TP53, TTN, and PIK3CA had the highest mutation frequencies, which might provide notable references for the investigation of BC (**Figures 7C, D**).

Subsequently, four drugs, including AZD1332_1463, BMS.754807_2171, mitoxantrone_1810, and nutlin.3a…._1047 had significantly positive correlations with the risk score (**Figure 7E**). These four drugs all had higher IC_50_ values in the HRG than in the LRG, implying that the tumor cells in the HRG had lower sensitivity to these drugs (**Figure 7F**). Spearman correlation analysis of prognostic gene expression levels versus Vinorelbine IC50 values revealed that among the 8 prognostic genes, 7 showed significant correlations: BRCA1 exhibited a positive correlation with Vinorelbine IC50 values, while IL7 demonstrated a negative correlation (**Figure 7G**).

This indicated that patients in the HRG might benefit less from these specific therapies, highlighting the importance of personalized treatment strategies based on risk stratification.

### 3.7 Macrophage was identified as a crucial cell for BC development

**Figure 8:**
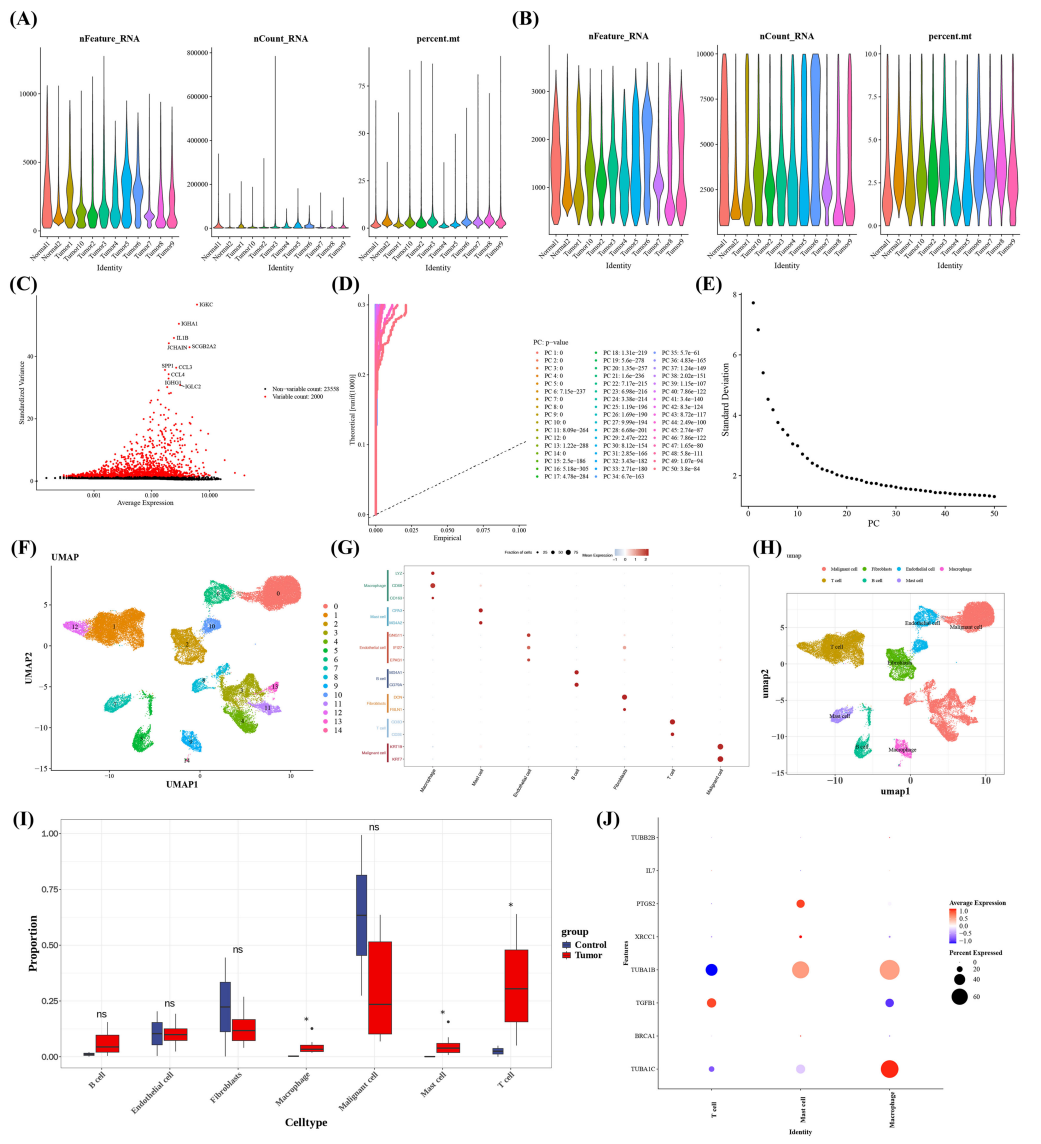
Single-cell RNA sequencing analysis identifies macrophages as key cells in breast cancer. **(A, B)** Quality-control results of single-cell RNA sequencing data. **(C)** Highly variable genes selected for downstream analysis. **(D, E)** Principal component analysis of single-cell data. **(F)** Clustering of 15 cell clusters. **(G, H)** Annotation of seven major cell types based on marker-gene expression. **(I)** Comparison of major cell-type abundance between breast cancer and normal samples. **(J)** Identification of macrophages as key cells for downstream analyses. *P < 0.05, and ns indicates no statistically significant difference.

First, a total of 44,732 eligible cells and 25,558 eligible genes were left from 69,275 cells and 25,558 genes (**Figures 8A, B**). Subsequently, 2,000 genes with the highest variation were chosen, such as IGKC and IGHA1, which showed the highest variation and might play important roles in subsequent analyses (**Figure 8C**). Furthermore, the first 30 PCs were subjected to carry out cell clustering (**Figures 8D, E**; **Supplementary Figure 1**), and 15 cell clusters were then clustered (**Figure 8F**). Afterward, seven cell types were clearly annotated according to the expression of markers in each cell (**Figures 8G, H**; **Supplementary Figure 2**). For example, macrophages were annotated relied on the expression of LYZ, CD68, and CD163, while fibroblasts were annotated according to the expression of DCN and FBLN1 (**Table 4**). These findings laid a solid foundation for further exploration of the cellular-level mechanisms underlying BC. Among these seven cell types, macrophages, mast cells, and T cells had notably higher abundance in BC samples than in normal samples (**Figure 8I**). Considering its higher abundance and potential functional significance in BC-related processes (such as its role in immune response and interaction with prognostic genes), macrophages were identified as crucial cells for subsequent analyses (**Figure 8J**).

**Table 4:** Marker genes used for cell-type annotation in single-cell RNA sequencing analysis of breast cancer.

Cell type	Marker gene
Macrophage	“LYZ”,”CD68”,”CD163”
Mast cell	“CPA3”, “MS4A2”
B cell	“MS4A1”, “CD79A”
Fibroblasts	“DCN”,”FBLN1”
Malignant cell	“KRT19”,”KRT7”
T cell	“CD3D”, “CD3E”
Endothelial cell	“GNG11”, “IFI27”,”EPAS1”

### 3.8 Spatial transcriptomic validation of TGFB1-associated macrophage and stromal niches

**Figure 9:**
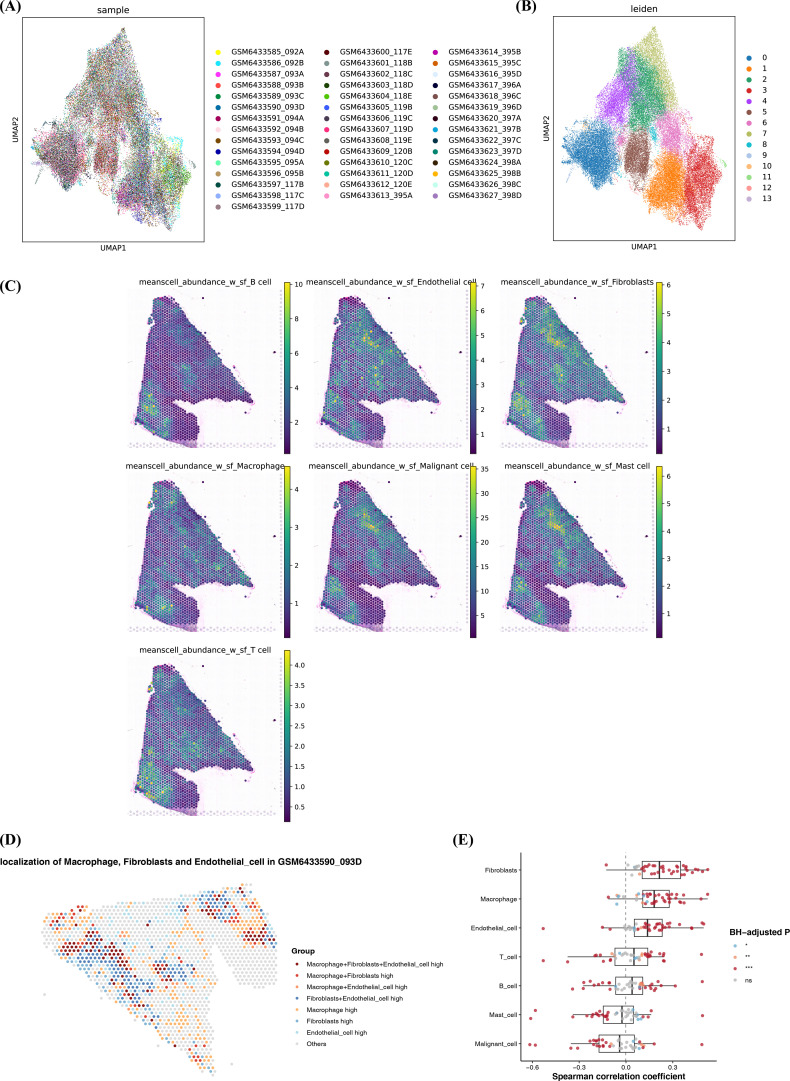
Spatial transcriptomic validation of TGFB1 expression and cellular niches in TNBC microenvironment. **(A)** UMAP visualization of integrated spatial transcriptomic data colored by sample origin. **(B)** UMAP visualization colored by Leiden clustering (resolution=0.5). **(C)** Representative spatial distribution of major cell populations across 55,670 spots from 42 TNBC samples. **(D)** Spatial co-localization analysis. **(E)** Spatial correlation analysis of TGFB1 expression with major cell populations. *P < 0.05, **P < 0.01, ***P < 0.001, and ns indicates no statistically significant difference.

To further validate the spatial relationship between TGFB1-related signals, macrophages, and the tumor microenvironment, we analyzed the publicly available TNBC spatial transcriptomics dataset GSE210616. The integrated spatial transcriptomic analysis was visualized via UMAP. The **Figure 9A**, colored by sample, showed specific clusters for TD1 and TD2 while the remaining samples were well-mixed, indicating successful batch effect removal by Harmony integration. The **Figure 9B**, colored by Leiden clustering (resolution = 0.5), identified 9 distinct and structurally independent clusters, demonstrating that the integrated data effectively retained biologically relevant subpopulation features. Cell2location was used to infer the relative abundance of major cell populations across 55,670 spatial spots from 42 samples. Malignant cells represented the dominant cellular component in TNBC tissues, while immune and stromal populations, including macrophages, fibroblasts, endothelial cells, T cells, B cells, and mast cells, exhibited marked spatial heterogeneity (**Table 5**, **Figure 9C** [Representative sample GSM6433590_093D]).

**Table 5:** Cell type and related data.

Cell type	Average abundance	Average relative composition (%)
Malignant cell	10.83	49.18
Mast cell	2.21	10.03
B cell	2.19	10.09
T cell	2.12	9.53
Macrophage	1.81	8.4
Endothelial cell	1.56	7.3
Fibroblasts	1.14	5.47

Spatial co-localization analysis showed that macrophage-enriched regions partially overlapped with fibroblast- and endothelial cell-enriched regions in representative sections, suggesting the presence of a macrophage-stromal-vascular niche within the TNBC microenvironment (**Figure 9D**). Furthermore, TGFB1 expression showed positive spatial correlations with fibroblasts, macrophages, and endothelial cells across samples, with mean Spearman correlation coefficients of 0.225, 0.184, and 0.140, respectively. In contrast, TGFB1 displayed a weak negative correlation with malignant cell abundance (-0.069) (**Table 6**, **Figure 9E**). These findings suggest that TGFB1-related signals are preferentially associated with stromal and immune-enriched regions rather than malignant cell-dominant regions.

**Table 6:** Summary of the spatial correlation between TGFB1 abundance and different cell types.

Cell type	Average Spearman r	Medium Spearman r	Number of samples with a significant positive correlation	Number of samples with a significant negative correlation
Fibroblasts	0.225	0.214	34	1
Macrophage	0.184	0.181	34	4
Endothelial cell	0.14	0.138	30	3
T cell	0.032	0.051	19	11
B cell	0.024	0.039	18	11
Mast cell	-0.06	-0.026	8	19
Malignant cell	-0.069	-0.041	8	18

Macrophage-related signature analysis further supported these observations (**Table 7**). The Macrophage_signature_score was strongly correlated with macrophage abundance (0.473), while the SPP1_CD44_Mac_like_score was positively associated with macrophage (0.465) and fibroblast (0.245) abundance, suggesting a stromal remodeling-associated macrophage-like state. In contrast, the CD80_CCL7_Mac_like_score was positively correlated with macrophage (0.308), T-cell (0.204), and Endothelial cell (0.148) abundance, indicating a potential immune activation or chemotaxis-associated macrophage spatial niche.

**Table 7:** Key spatial correlation findings of the macrophage-associated signature.

Relevant cell types	Average Spearman r	Medium Spearman r	Number of samples with a significant positive correlation	Number of samples with a significant negative correlation
Macrophage	0.473	0.51	39	2
Fibroblasts	0.359	0.421	38	3
Endothelial cell	0.084	0.107	23	10
Macrophage	0.465	0.497	41	0
Fibroblasts	0.245	0.285	36	4
Endothelial cell	0.12	0.129	26	9
Macrophage	0.308	0.294	41	0
T cell	0.204	0.181	32	2
Endothelial cell	0.148	0.143	30	4

### 3.9 TGFB1-centered crosstalk in the tumor immune microenvironment.

**Figure 10:**
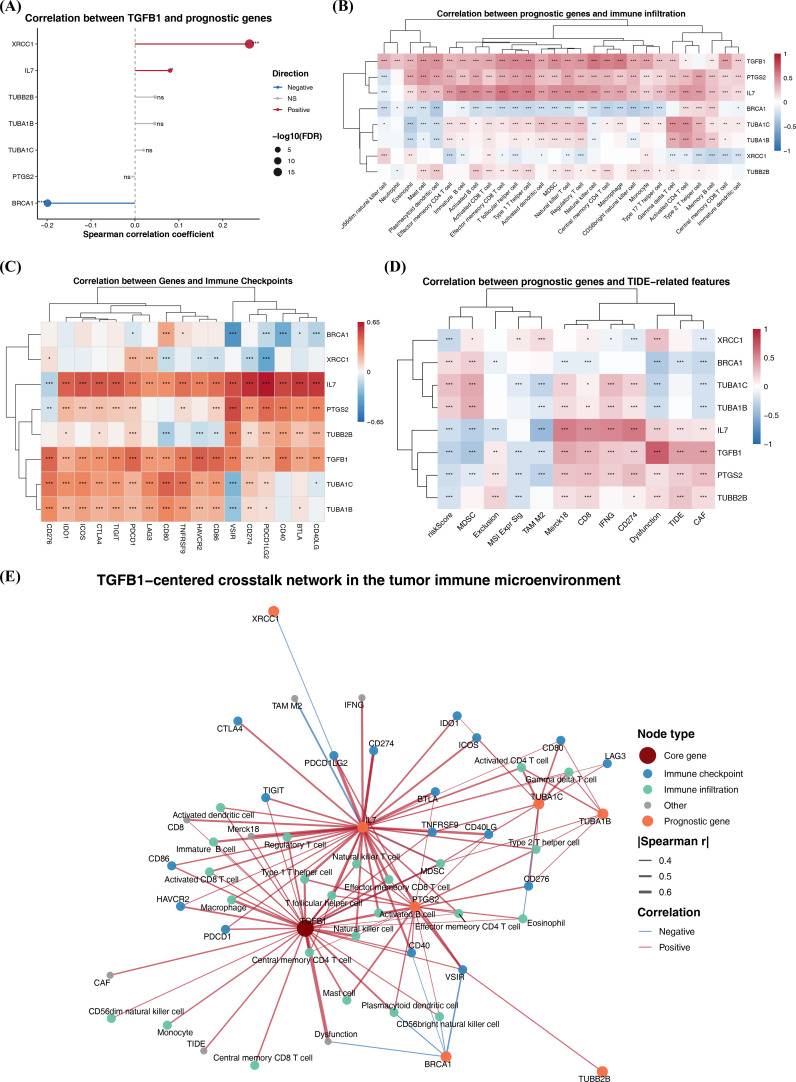
TGFB1-centered crosstalk in the tumor immune microenvironment. **(A)** Correlation analysis between TGFB1 and other prognostic genes. **(B)** Correlation between TGFB1 and immune infiltration features. **(C)** Correlation between TGFB1 and immune checkpoint molecules. **(D)** Correlation between TGFB1 and TIDE-related indicators. **(E)** TGFB1-centered crosstalk network containing 116 edges (60 immune infiltration edges, 41 checkpoint edges, 15 TIDE edges). *P < 0.05, **P < 0.01, ***P < 0.001, and ns indicates no statistically significant difference.

We systematically evaluated the crosstalk between TGFB1 and other prognostic genes, immune features, checkpoint molecules, and TIDE indicators. TGFB1 was significantly positively correlated with XRCC1 (r = 0.261, FDR = 1.46e-19) and IL7 (r = 0.080, FDR = 0.011), and negatively correlated with BRCA1 (r = -0.199, FDR = 8.82e-12); no direct significant correlations were observed with TUBA family genes or PTGS2 (all FDR > 0.05, **Table 8**, **Figure 10A**). TGFB1 showed strong positive correlations with immune infiltration features including macrophages (r = 0.560), natural killer cells (r = 0.580), central memory CD4 T cells (r = 0.522), and effector memory CD8 T cells (r = 0.484) (all FDR < 0.001, **Table 9**; **Figure 10B**). It was significantly positively correlated with 17 immune checkpoint molecules, with the strongest associations for HAVCR2 (r = 0.454), CD276 (r = 0.429), and PDCD1 (r = 0.419) (all FDR < 0.001, **Table 10**; **Figure 10C**). Regarding TIDE-related indicators, TGFB1 exhibited an extremely strong positive correlation with T-cell dysfunction score (r = 0.697, FDR = 1.27e-155) and was positively correlated with TIDE total score (r = 0.413), CAF (r = 0.409), and CD8 infiltration (r = 0.311) (all FDR < 0.001, **Table 11**, **Figure 10D**). A TGFB1-centered crosstalk network was constructed containing 116 edges (60 immune infiltration edges, 41 checkpoint edges, 15 TIDE edges), confirming TGFB1 as a highly connected hub linking multiple immune-stromal components (**Figure 10E**).

**Table 8:** Correlation between TGFB1 expression and other prognostic genes.

Gene	Spearman r	FDR	Direction
TGFB1	1	<1e-300	positive correlation
XRCC1	0.261	1.46E-19	positive correlation
BRCA1	-0.199	8.82E-12	negative correlation
IL7	0.08	0.011	positive correlation
TUBB2B	0.046	0.157	no significant
TUBA1B	0.045	0.157	no significant
TUBA1C	0.02	0.567	no significant
PTGS2	-0.005	0.852	no significant

**Table 9:** The common association between prognostic genes and immune infiltration characteristics.

TGFB1-related immune characteristics	Spearman r	FDR	Significance
Natural killer cell	0.58	1.09E-97	***
Macrophage	0.56	6.06E-90	***
Central memory CD4 T cell	0.522	1.78E-76	***
T follicular helper cell	0.511	8.75E-73	***
Type 1 T helper cell	0.508	7.81E-72	***
MDSC	0.49	3.7E-66	***
Effector memeory CD8 T cell	0.484	1.82E-64	***
Plasmacytoid dendritic cell	0.472	3.94E-61	***
Regulatory T cell	0.45	6.79E-55	***
Mast cell	0.441	1.05E-52	***

***P < 0.001.

**Table 10:** Correlation between prognostic genes and immune checkpoint molecules.

TGFB1-related checkpoint	Spearman r	FDR	Significance
HAVCR2	0.454	6.06E-61	***
CD276	0.429	4.62E-54	***
CD86	0.421	5.18E-52	***
PDCD1	0.419	9.23E-52	***
TNFRSF9	0.373	1.71E-40	***
CD40	0.37	5.73E-40	***
VSIR	0.364	1.58E-38	***
TIGIT	0.345	1.17E-34	***
CTLA4	0.3	3.86E-26	***
ICOS	0.291	1.27E-24	***

***P < 0.001.

**Table 11:** Correlation between prognostic genes and TIDE-associated immunotherapy indicators.

TIDE index of correlation	Spearman r	FDR	Significance	Direction
Dysfunction	0.697	1.27E-155	***	Positive
TIDE	0.413	1.18E-44	***	Positive
CAF	0.409	6.72E-44	***	Positive
Merck18	0.373	2.34E-36	***	Positive
CD8	0.311	3.2E-25	***	Positive
IFNG	0.231	3.11E-14	***	Positive
CD274	0.213	2.65E-12	***	Positive
Exclusion	0.09	0.003	**	Positive
MSI Expr Sig	-0.185	1.01E-09	***	Negative
TAM M2	-0.19	3.9E-10	***	Negative
riskScore	-0.299	2.36E-23	***	Negative
MDSC	-0.373	2.34E-36	***	Negative

**P < 0.01, ***P < 0.001.

### 3.10 Communication types and expression trend of prognostic genes in macrophage

**Figure 11:**
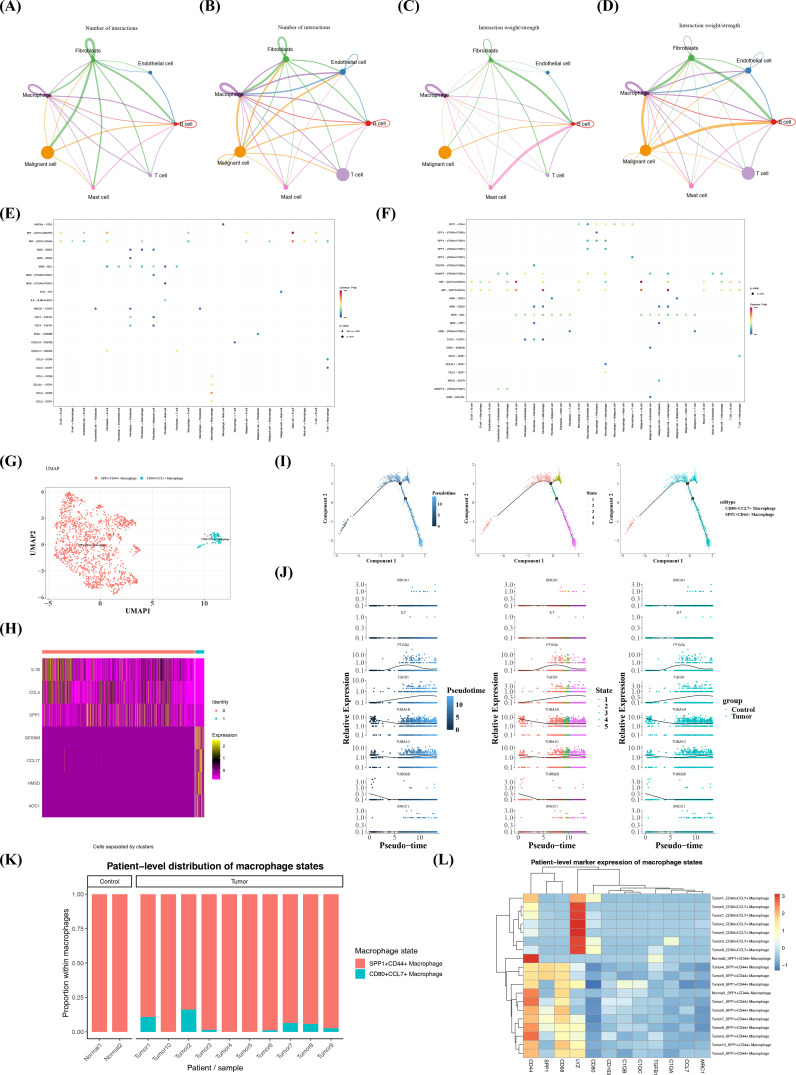
Intercellular communication and differentiation trajectory of macrophages in breast cancer. **(A–D)** Number and strength of cell–cell interactions involving macrophages. **(E, F)** MIF-related ligand–receptor communication between macrophages and other cell types. **(G)** Identification of two macrophage subclusters. **(H)** Expression patterns of prognostic genes in macrophage subclusters. **(I)** Pseudotime distribution of macrophage differentiation states. **(J)** Dynamic expression of prognostic genes during macrophage differentiation. **(K)** Patient-level distribution of SPP1^+^CD44^+^ and CD80^+^CCL7^+^ macrophage states in breast cancer. **(L)** Heatmap of marker expression for SPP1^+^CD44^+^ and CD80^+^CCL7^+^ macrophages across samples highlighting patient-level heterogeneity.

As per the results of cell-cell communication analysis, the number and strength of interactions between macrophages and other cell types all increased (**Figures 11A–D**). Specifically, the number of interactions between macrophages and fibroblasts increased, the interactions between macrophages and endothelial cells were activated, and the strength of interactions between macrophages and malignant cells was strengthened. Moreover, MIF-(CD74^+^CXCR4^+^) and MIF-(CD74^+^CD44^+^) might play notable roles in mediating communication between macrophages and other cell types (**Figures 11E, F**; **Supplementary Figure 3**). Subsequently, macrophages were further annotated as two cell subgroups (SPP1^+^CD44^+^macrophages and CD80^+^CCL7^+^macrophages), in which SPP1^+^CD44^+^macrophages were more abundant (**Figure 11G**). For prognostic genes, three (IL1B, CCL4, and SPP1) were detected in SPP1^+^CD44^+^macrophages, while four were mainly expressed in CD80^+^CCL7^+^macrophages (**Figure 11H**). Furthermore, the whole differentiation process of macrophages was classified into five states. CD80^+^CCL7^+^macrophages were annotated in the late stage of differentiation (state 1), and SPP1^+^CD44^+^macrophages were mainly annotated in the early stages of differentiation (states 2, 3, 4, and 5) (**Figure 11I**; **Supplementary Figure 4**). In the whole differentiation process of macrophages, the expression level of TGFB1 increased, the expression of TUBA1B and TUBB2B decreased in the early stage of differentiation, the expression of TUBA1C was stable, and PTGS2 was mainly detected in the middle stage of differentiation (**Figure 11J**). SPP1^+^CD44^+^ macrophages stably present across all samples and predominantly enriched, indicating a ubiquitous, stable state in breast cancer. Conversely, CD80^+^CCL7^+^macrophages specifically enriched in subsets, reflecting inflammatory/activation states. They exhibit heterogeneous distribution patterns among patients (**Figures 11K,L**).

### 3.11 Experimental validation of the antitumor efficacy and immune modulation of VLB plus anti-PD-1 therapy

#### 3.11.1 VLB plus anti-PD-1 combination therapy exhibits superior antitumor efficacy *in vivo*

**Figure 12:**
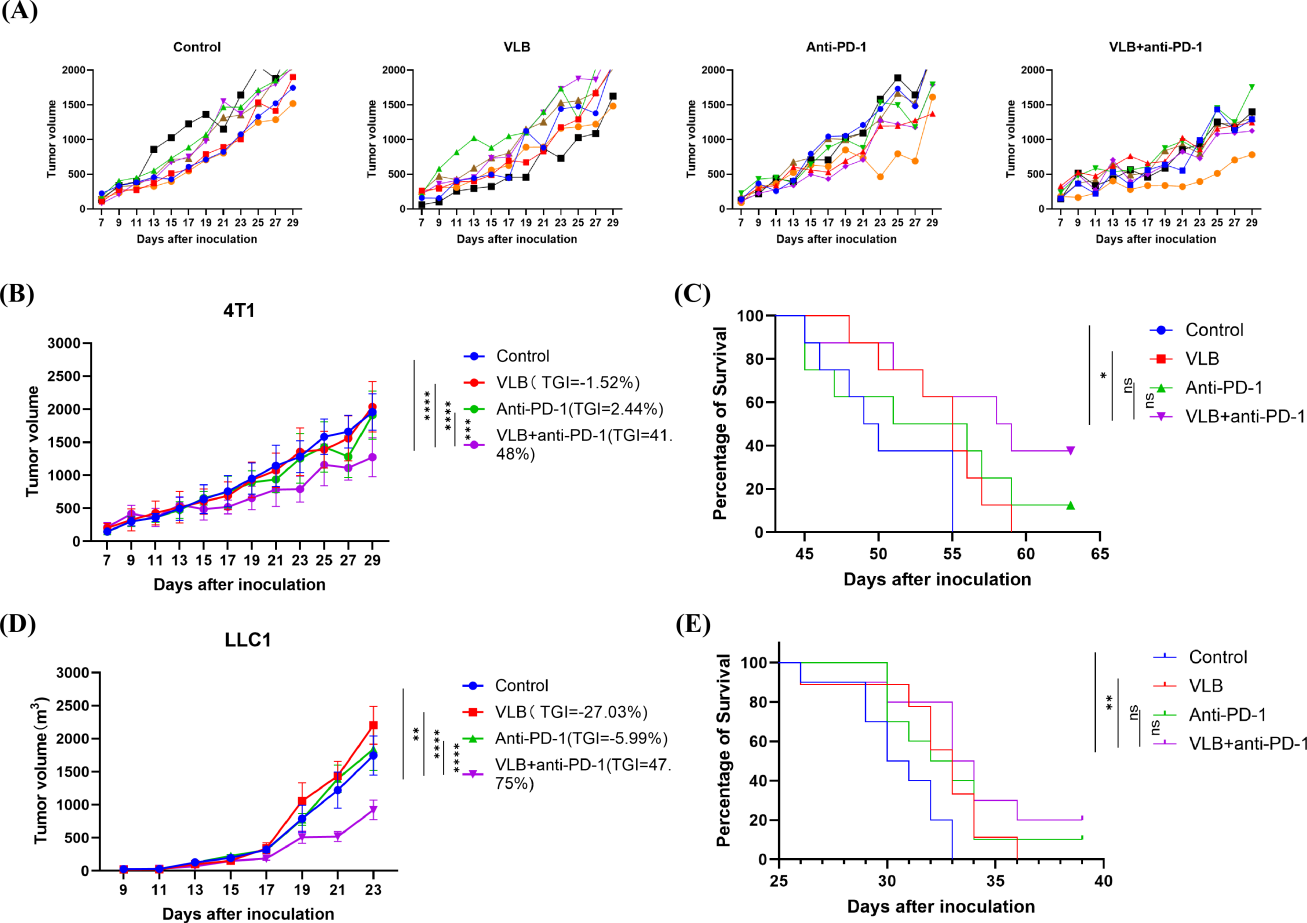
VLB plus anti-PD-1 combination therapy exhibits superior antitumor efficacy *in vivo*. **(A, B)** The 4T1 subcutaneous tumor model was established in BALB/c mice; individual and grouped tumor growth curves are shown, with n = 7 mice per group. **(C)** Overall survival of 4T1 tumor-bearing mice following treatment, with n = 8 mice per group. **(D, E)** The LLC1 subcutaneous tumor model was established in C57BL/6 mice. Tumor growth and survival analyses are shown, with n = 9–10 mice per group. Tumor growth data are presented as mean ± SD and were analyzed using ordinary two-way ANOVA. Survival differences were analyzed using the Kaplan–Meier method with log-rank (Mantel–Cox) test. **P < 0.01, ***P < 0.001, ****P < 0.0001, and ns indicates no statistically significant difference.

Both 4T1 BC and LLC1 murine tumor models were established and subjected to different treatment regimens, including control, vinorelbine (VLB) monotherapy, anti-PD-1 monotherapy, and VLB plus anti-PD-1 combination therapy. In the 4T1 tumor model, tumor growth was moderately inhibited by either VLB or anti-PD-1 monotherapy, whereas the combination treatment markedly suppressed tumor progression compared with all other groups (**Figures 12A,B**). Quantitative analysis showed that the tumor growth inhibition (TGI) rate of the combination group reached 41.48%, which was substantially higher than that observed in the monotherapy groups, indicating a synergistic antitumor effect of VLB combined with immune checkpoint blockade. Although no statistically marked difference in overall survival was observed among the treatment cohorts in one experimental batch of the 4T1 model (**Supplementary Figure 5A**), a significant survival benefit of the combination therapy was observed in an independent experimental batch (**Figure 12C**). Consistently, this batch also exhibited robust tumor growth suppression in the VLB plus anti-PD-1 group (**Supplementary Figures 5C,D**), supporting the reproducibility of the antitumor efficacy of the combination regimen despite inter-experimental variability.

To further validate these findings in a distinct tumor context, a subcutaneous LLC1 tumor model was established. Similar to the results observed in the 4T1 model, VLB plus anti-PD-1 treatment significantly inhibited tumor growth, with a TGI rate of 47.75%, which was markedly higher than that of either monotherapy (**Figure 12D**; **Supplementary Figure 5E**). Notably, the combination therapy also resulted in a significant prolongation of survival in LLC1 tumor-bearing mice compared with the control and single-agent treatment groups (**Figure 12E**). Importantly, no obvious body-weight loss was probe in any treatment group throughout the experimental period in both tumor models (**Supplementary Figures 5B, F**), suggesting that the combination therapy was well tolerated and did not induce overt systemic toxicity.

Collectively, these *in vivo* results demonstrate that vinorelbine combined with anti-PD-1 therapy exerts superior antitumor efficacy compared with monotherapy, effectively suppressing tumor growth and improving survival in multiple murine tumor models, thereby providing a solid experimental basis for further investigation of its underlying immunomodulatory mechanisms.

#### 3.11.2 Vinorelbine modulates macrophage phenotype and cytokine production *in vitro*

Given that the bioinformatic and single-cell analyses identified macrophages as crucial modulators of the BC immune microenvironment, *in vitro* experiments were further conducted to appraise the direct effects of vinorelbine on macrophage phenotype and cytokine production. BMDMs were generated from murine bone marrow cells following stimulation with macrophage colony-stimulating factor (M-CSF) (**Supplementary Figure 6A**). Upon additional stimulation with interleukin-4 (IL-4), the proportion of M2-like macrophages increased, accompanied by elevated expression of the M2-associated marker CD206 and the M1-associated marker CD86, confirming successful macrophage polarization under the experimental conditions (**Supplementary Figures 6B,C**).

**Figure 13:**
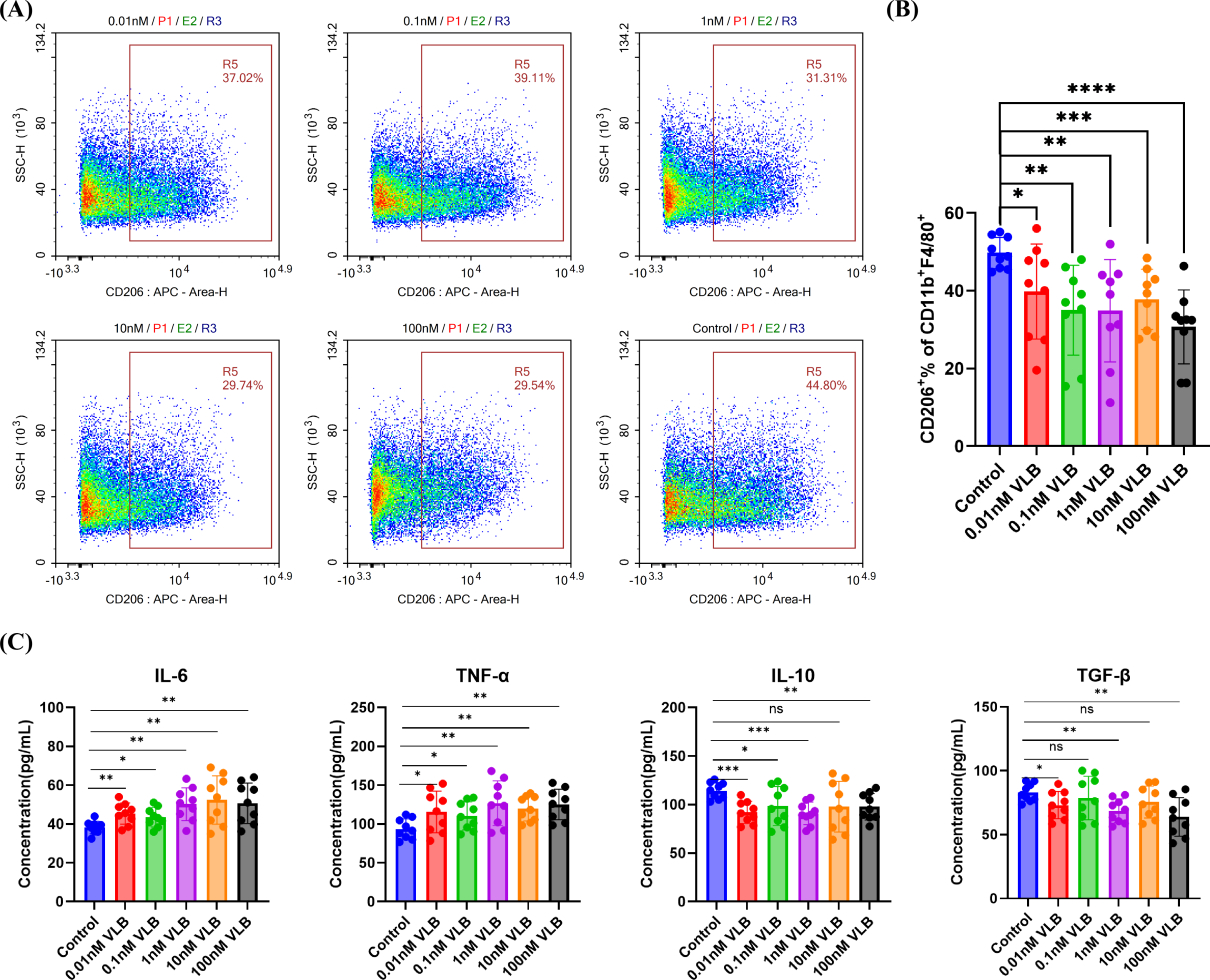
Different concentrations of VLB induce polarization of bone marrow–derived macrophages from the M2 phenotype toward the M1 phenotype. **(A, B)** CD206, an M2-associated marker, was downregulated following treatment with different concentrations of VLB. Representative flow-cytometry plots **(A)** and quantitative analysis **(B)** are shown. Data were obtained from three independent experiments, with a total of 9 data points per group. Data are presented as mean ± SD from three independent experiments with three replicates per experiment (n = 9 per group) and were analyzed using an unpaired two-tailed t test. **(C)** Enzyme-linked immunosorbent assay showing changes in IL-6, TNF-α, IL-10, and TGF-β levels in cell-culture supernatants after treatment with different concentrations of VLB. Data are presented as mean ± SD from three independent experiments with three replicates per experiment (n = 9 per group) and were analyzed using an unpaired two-tailed t test. *P < 0.05, **P < 0.01, ***P < 0.001, ****P < 0.0001, and ns indicates no statistically significant difference.

IL-4–stimulated BMDMs were treated with increasing concentrations of vinorelbine. Flow cytometric analysis revealed that vinorelbine treatment found in a dose-dependent reduction in the fraction of CD206^+^ cells within the CD11b^+^F4/80^+^ macrophage population (**Figures 13A,B**). Compared with the control group, the frequency of CD206^+^ macrophages was markedly decreased following vinorelbine exposure, indicating an alteration in macrophage phenotypic composition. In parallel, culture supernatants were collected for cytokine quantification by ELISA. The results showed that the concentrations of the pro-inflammatory cytokines IL-6 and TNF-α were increased following vinorelbine treatment, whereas the levels of the IL-10 and TGF-β were reduced, compared with the control group (**Figure 13C**). These changes were consistently observed across different vinorelbine concentrations. Taken together, the flow cytometric and cytokine profiling data demonstrate that vinorelbine treatment alters macrophage phenotypic markers and cytokine secretion profiles *in vitro*. These findings are consistent with our single-cell analyses identifying macrophages as key modulators of the tumor immune microenvironment.

#### 3.11.3 RT-qPCR analysis reveals immune-related transcriptional changes in tumor tissues

**Figure 14:**
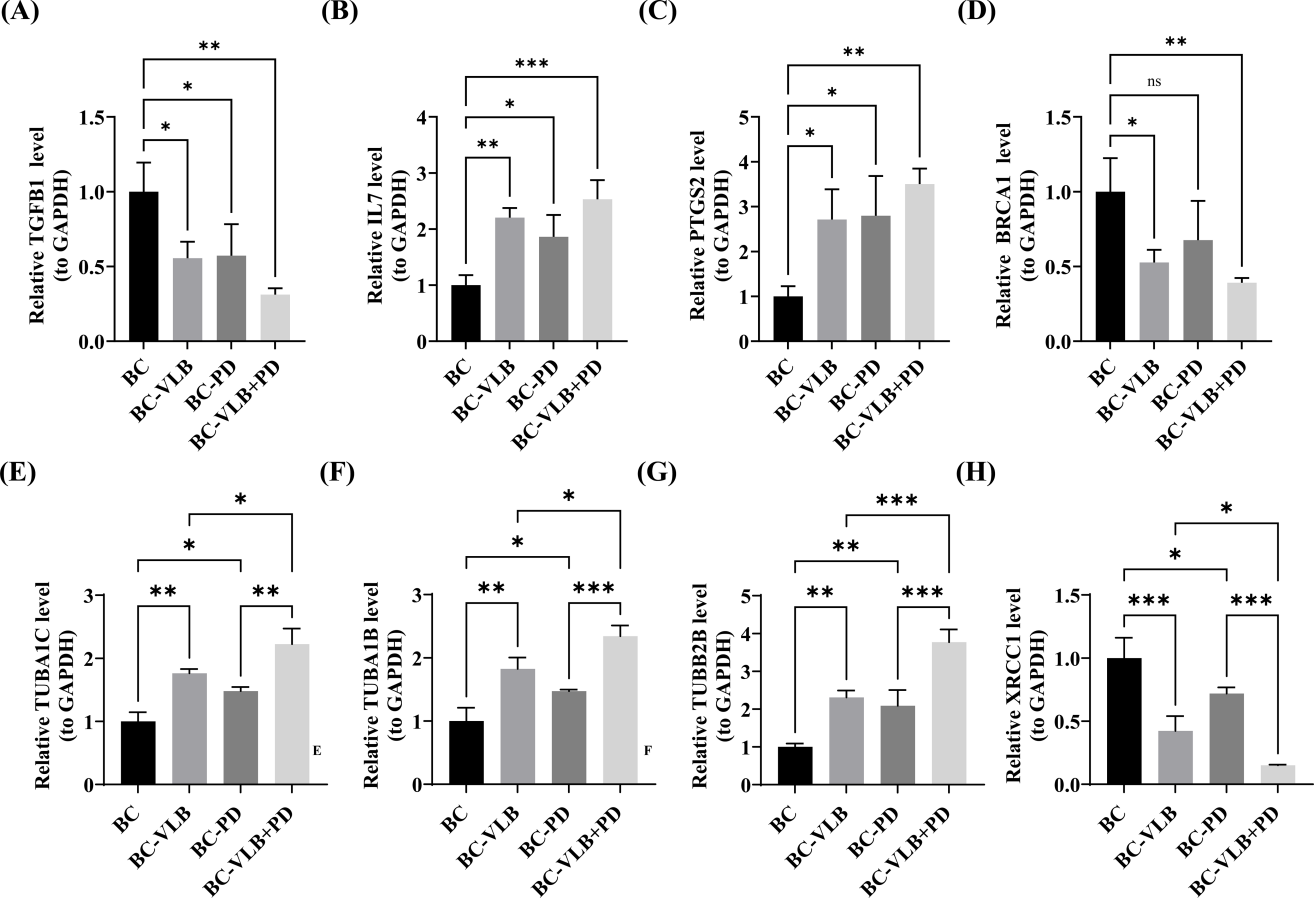
RT-qPCR validation of prognostic gene expressions in breast cancer tissues. The mRNA expression levels of **(A)** *TGFB1*, **(B)** *IL7*, **(C)** *PTGS2*, **(D)** *BRCA1*, **(E)** *TUBA1C*, **(F)** *TUBA1B*, **(G)** *TUBB2B* and **(H)** *XRCC1* were quantified by RT-qPCR in four groups: BC (untreated breast cancer), BC-VLB (Vinorelbine treated), BC-PD (PD-1 inhibitor treated), and BC-VLB+PD (combination therapy). GAPDH was used as an internal control for normalization. Data are presented as mean ± SD. *P < 0.05, **P < 0.01, ***P < 0.001, and ns indicates no statistically significant difference.

RT-qPCR results revealed markedly elevated TGFB1 expression in BC specimens compared to the control cohort (P < 0.05), suggesting its potential as a biomarker for disease progression. Notably, TGFB1 expression was significantly lower in BC-VLB + PD samples (treated with both Vinorelbine and PD), suggesting that the combination therapy of VLB and PD may effectively inhibit TGFB1 expression and thus be beneficial for treating BC (**Figure 14A**). This *in vivo* transcriptional change was consistent with the reduced TGF-β levels observed in vinorelbine-treated macrophage culture supernatants *in vitro*, as described in Section 3.9.2. For IL7 and PTGS2, their expressions were significantly lower in BC samples but significantly higher in BC-VLB + PD samples (P < 0.05) (**Figures 14B,C**). This finding implies that these genes may be involved in the immune response and inflammation pathways, and their upregulation in the combination therapy group might reflect a more favorable immune environment for cancer treatment. Additionally, BRCA1 expression showed significant differences between BC samples and both BC-VLB and BC-VLB + PD samples (P < 0.05). Notably, BRCA1 expression was markedly higher in BC samples, which is in agreement with its known role in DNA repair and genomic stability. This suggests that BRCA1 might influence the response to chemotherapy and targeted therapy in BC (**Figure 14D**). Compared with the BC group, the BC-VLB, BC-PD, and BC-VLB+PD groups all showed significantly higher expression of TUBA1C, TUBA1B, and TUBB2B (P<0.05) and significantly lower expression of XRCC1 (P < 0.05). Compared with the BC-VLB group, the BC-VLB+PD group exhibited further upregulation of TUBA1C, TUBA1B, and TUBB2B (P < 0.05) and further downregulation of XRCC1 (P < 0.05). Similarly, compared with the BC-PD group, the BC-VLB+PD group showed significantly higher expression of TUBA1C, TUBA1B, and TUBB2B (P < 0.05) and significantly lower expression of XRCC1 (P < 0.05) (**Figures 14E–H**). Together, these transcriptional changes at the tumor tissue level provide molecular support for the immune-modulatory effects suggested by *in vitro* macrophage assays, laying a foundation for advancing vinorelbine plus anti-PD-1 combination therapy into prospective clinical trials.

## 4 Discussion

BC is a highly complex and heterogeneous disease, exhibiting substantial intertumoral and intratumoral heterogeneity, which leads to marked variability in therapeutic responses and clinical outcomes (3, 4). We integrated data from multiple public databases, including TCGA and GEO, and applied a series of comprehensive bioinformatics analyses to identify eight prognostic genes (TUBA1C, BRCA1, TGFB1, TUBA1B, XRCC1, PTGS2, IL7, and TUBB2B). Based on these genes, we constructed a prognostic risk model and a nomogram, both of which demonstrated robust predictive performance. Furthermore, we systematically investigated the tumor immune microenvironment, enriched signaling pathways, tumor mutational burden (TMB), drug sensitivity, and single-cell characteristics. Collectively, our findings provide novel insights into prognostic evaluation and therapeutic strategies for BC.

Among the identified prognostic genes, microtubule-associated genes, including TUBA1C, TUBA1B, and TUBB2B, emerged as key risk factors, consistent with the known mechanism of vinorelbine as a microtubule-disrupting agent. Aberrant expression of tubulin family members has been widely reported to promote tumor cell proliferation, mitotic instability, and therapeutic resistance in multiple malignancies, including BC (43, 44). Previous studies have demonstrated that elevated TUBA1C expression enhances tumor aggressiveness by activating oncogenic signaling pathways and promoting cytoskeletal remodeling in lung and ovarian cancers, which aligns with our observation that these genes contribute to poor prognosis in BC (45–47). These findings suggest that vinorelbine-related modulation of microtubule dynamics may influence not only tumor cell division, but also downstream biological processes associated with disease progression.

BRCA1, a well-established tumor suppressor gene involved in DNA damage repair and genomic stability, also showed strong prognostic relevance in our model. Consistent with previous reports, BRCA1 dysregulation was associated with altered survival outcomes in BC patients (48, 49). Although BRCA1 is classically considered a protective factor, its expression patterns and functional roles may vary depending on tumor subtype, treatment context, and immune microenvironment (50, 51). Our findings further support the notion that BRCA1 status may influence therapeutic responses, particularly in the context of chemotherapy and combination treatment strategies.

Transforming Growth Factor Beta 1 (TGFB1) is a multifunctional cytokine that plays an exceptionally complex role in tumorigenesis, exhibiting pronounced dual functional properties. During the early stages of cancer development, TGFB1 primarily exerts anti-proliferative effects. It activates the TGFB1–Smad signaling pathway, thereby inducing the expression of cyclin-dependent kinase inhibitors (CKIs), such as p15 and p21. These CKIs suppress the activity of cyclin-dependent kinases (CDKs), ultimately inhibiting cancer cell proliferation (52–54). Notably, TGFB1 emerged as a central immune-related prognostic gene in our analysis. In our bulk transcriptomic and immune infiltration analyses, TGFB1 expression was directly correlated with multiple immune cell populations and risk scores, suggesting its involvement in shaping an immunosuppressive TME. Importantly, single-cell analysis revealed a progressive increase in TGFB1 expression along macrophage differentiation trajectories, further implicating macrophages as a major cellular source and functional mediator of TGFB1 signaling in BC.

Spatial transcriptomic analysis further validated the distribution pattern of TGFB1 signaling: TGFB1 expression showed significant positive correlations with fibroblasts, macrophages, and endothelial cells, but a weak negative correlation with malignant cells, indicating that TGFB1-related signals are preferentially localized in stromal and immune-enriched regions rather than malignant cell-dominated areas. Additionally, spatial co-localization analysis revealed partial overlaps between macrophage-enriched regions and fibroblast/endothelial cell-enriched regions, forming a macrophage-stromal-vascular niche, supporting the regulatory role of TGFB1 in this niche.

The consistency between bioinformatic predictions and experimental validation strengthens this conclusion. In the present study, our experimental findings further substantiated the bioinformatic observations. *In vitro*, vinorelbine treatment of BMDMs led to reduced secretion of TGF-β and a shift in cytokine profiles toward a pro-inflammatory phenotype, as evidenced by increased IL-6 and TNF-α levels. Consistently, our *in vivo* results provided additional validation at the tumor tissue level. *In vivo*, RT-qPCR analysis of tumor tissues from vinorelbine plus anti-PD-1–treated mice demonstrated significantly decreased TGFB1 expression compared with untreated controls. This concordance across single-cell analysis, macrophage functional assays, and tumor-level transcriptional validation provides multi-layered evidence supporting the role of macrophage-associated TGFB1 modulation in vinorelbine-mediated immune regulation. Moreover, comparable associations between macrophage-derived TGF-β signaling and tumor immune suppression have been reported in BC and other solid tumors (55, 56), further supporting the biological plausibility of our findings.

Beyond TGFB1, other immune-related prognostic genes, including PTGS2 and IL7, also exhibited treatment-associated transcriptional changes. PTGS2 (COX-2) is a key mediator of inflammation and prostaglandin synthesis, and its dysregulation has been linked to tumor-associated inflammation, angiogenesis, and immune suppression in BC (57, 58). IL7 plays a crucial role in T-cell survival and immune homeostasis, and alterations in IL7 signaling have been associated with antitumor immune responses and immunotherapy efficacy (59, 60). The upregulation of IL7 and PTGS2 observed in the combination therapy group may reflect dynamic immune remodeling in response to vinorelbine-based treatment, although the precise mechanisms require further investigation.

Our immune microenvironment analyses further revealed that macrophages, along with T cells and mast cells, were markedly enriched in BC tissues and closely associated with risk stratification. Cell–cell communication analysis highlighted enhanced interactions between macrophages and malignant cells, and fibroblasts and endothelial cells, mediated in part by MIF–CD74 signaling axes. These findings are consistent with previous studies demonstrating that macrophage-centered communication networks play pivotal roles in tumor progression and immune regulation (55, 61). Importantly, our experimental data suggest that vinorelbine may partially reprogram macrophage phenotypes, providing a potential explanation for the enhanced antitumor efficacy observed with vinorelbine plus anti-PD-1 combination therapy *in vivo*.

The crosstalk analysis reveals that TGFB1 is not an isolated prognostic marker, but a central hub embedded in an immune-stromal regulatory network. Its associations with XRCC1/BRCA1, macrophage infiltration, checkpoint activation, and T-cell dysfunction collectively shape the tumor microenvironment, potentially influencing patient prognosis and the biological significance of vinorelbine-related candidate genes. These findings align with previous studies showing TGF-β promotes immune suppression and stromal remodeling (62) and contributes to immunotherapy resistance (63). However, our study is based on correlation analyses, which cannot directly prove causal regulation of these processes by TGFB1. Future functional experiments (e.g., TGFB1 knockdown/overexpression *in vitro* and *in vivo*) are needed to validate these mechanisms. Additionally, the TIDE algorithm’s inferred MDSC/TAM M2 metrics may reflect distinct myeloid subsets, so we interpret TGFB1’s association with macrophage/myeloid remodeling rather than simple M2 macrophage accumulation.

Building upon this observation, increasing evidence indicates that vinorelbine exerts immunomodulatory effects beyond its direct cytotoxic activity, particularly through regulating macrophage function and polarization (8, 11, 64). Tumor-associated macrophages, especially M2-like macrophages, are known to promote tumor progression by fostering immunosuppression, angiogenesis, and tumor cell invasion. In this context, recent studies have demonstrated that vinorelbine can suppress M2 macrophage polarization and attenuate M2-associated immunosuppressive functions, thereby enhancing antitumor immune responses in non-small cell lung cancer (64). Consistent with these observations, our *in vitro* experiments showed that vinorelbine treatment reduced the proportion of CD206^+^ macrophages, accompanied by increased secretion of IL-6 and TNF-α. Together, these findings indicate that macrophage functional reprogramming represents an important immunological mechanism underlying vinorelbine-mediated antitumor activity, which may be conserved across different solid tumors.

Furthermore, GSEA revealed that 167 signaling pathways were markedly enriched between the HRG and LRG. These pathways are closely associated with tumor proliferation, immune regulation, and microenvironmental remodeling, suggesting that the prognostic signature may influence BC progression through coordinated modulation of cell cycle activity and immune-related processes. Notably, the cell cycle pathway represents a central regulatory network governing cellular proliferation by orchestrating the activity of cyclins, cyclin-dependent kinases (CDKs), and cyclin-dependent kinase inhibitors (CKIs), thereby controlling G1/S transition and G2/M progression. Dysregulation of cell cycle–related genes has been recognized as a fundamental driver of tumorigenesis and progression in BC (65, 66). Aberrant activation of this pathway not only promotes uncontrolled proliferation but also contributes to genomic instability and altered DNA damage responses (67). In BC, sustained cell cycle signaling has been associated with higher tumor grade, poor prognosis, and therapeutic resistance (68), and emerging evidence further indicates that cell cycle regulators may interact with immune and stress-response pathways to facilitate tumor progression (69). Consistently, the significant enrichment of the cell cycle pathway in the HRG supports the biological relevance of our prognostic model and suggests that enhanced proliferative signaling may underlie the unfavorable clinical outcomes observed in these patients.

In conclusion, this study establishes an integrative framework combining transcriptomic analysis, single-cell profiling, and experimental validation to identify vinorelbine-related prognostic genes in BC. Our findings highlight macrophage-associated TGFB1 modulation as a critical link between vinorelbine treatment and immune microenvironment remodeling, providing novel insights into prognostic assessment and potential combination therapeutic strategies.

However, this study still has limitations. First, the prognostic model was primarily developed and validated using retrospective public datasets, which may introduce selection bias and limit the generalizability of the findings. Additionally, the scRNA-seq analysis used the GSE245601 dataset (10 breast tumor, 2 normal samples)—a small cohort that may not fully capture breast cancer cellular heterogeneity. Therefore, prospective validation in larger, multi-center clinical cohorts is still required. Second, although our *in vivo* tumor models, *in vitro* macrophage stimulation assays, and RT-qPCR analyses collectively support the immunomodulatory effects of vinorelbine, direct evidence from prospective clinical trials is lacking to verify the efficacy of vinorelbine combined with anti-PD-1 therapy in patients stratified by the risk model. Further mechanistic investigations are necessary to delineate the complete causal signaling cascade connecting vinorelbine exposure, macrophage polarization, and TGFB1 regulation. Future work will include multiple experimental designs, for example, *in vitro* experiments using breast cancer-specific cell lines and *in vivo* studies with patient-derived xenograft models, which will not only systematically evaluate the expression dynamics of all eight prognostic genes upon vinorelbine exposure but also conduct knockdown/overexpression functional validation of candidate prognostic genes (e.g., TGFB1, BRCA1, tubulin family) to confirm their specific roles in vinorelbine treatment response. In addition, while single-cell analysis identified macrophages as key regulators within the TME, the potential contributions of other immune and stromal cell populations warrant further exploration.

Future studies should focus on validating our prognostic model in prospective clinical settings and dissecting the molecular mechanisms underlying vinorelbine-mediated immune modulation at higher resolution. A deeper understanding of macrophage–tumor interactions may facilitate the rational design of chemo-immunotherapy combination strategies for BC.

**Nomenclature**

## Data Availability

The datasets presented in this study can be found in online repositories. The names of the repository/repositories and accession number(s) can be found in the article/**Supplementary Material**.

## References

[1] Xiong X Zheng LW Ding Y Chen YF Cai YW Wang LP . Breast cancer: pathogenesis and treatments. Signal Transduct Tgt Ther. (2025) 10:49. doi: 10.1038/s41392-024-02108-4 39966355 PMC11836418

[2] Katsura C Ogunmwonyi I Kankam HK Saha S . Breast cancer: presentation, investigation and management. Br J Hosp Med Lond. (2022) 83:1–7. doi: 10.12968/hmed.2021.0459 35243878

[3] Li Y Zhao X Liu Q Liu Y . Bioinformatics reveal macrophages marker genes signature in breast cancer to predict prognosis. Ann Med. (2021) 53:1019–31. doi: 10.1080/07853890.2021.1914343 34187256 PMC8253219

[4] Grimaldi AM Nuzzo S Condorelli G Salvatore M Incoronato M . Prognostic and clinicopathological significance of MiR-155 in breast cancer: a systematic review. Int J Mol Sci. (2020) 21:5834. doi: 10.3390/ijms21165834 32823863 PMC7461504

[5] Teichgraeber DC Guirguis MS Whitman GJ . Breast cancer staging: updates in the AJCC cancer staging manual, 8th edition, and current challenges for radiologists, from the AJR special series on cancer staging. AJR Am J Roentgenol. (2021) 217:278–90. doi: 10.2214/ajr.20.25223 33594908

[6] Li Q Liu H Jin Y Yu Y Wang Y Wu D . Analysis of a new therapeutic target and construction of a prognostic model for breast cancer based on ferroptosis genes. Comput Biol Med. (2023) 165:107370. doi: 10.1016/j.compbiomed.2023.107370 37643511

[7] Dvir K Giordano S Leone JP . Immunotherapy in breast cancer. Int J Mol Sci. (2024) 25:7517. doi: 10.3390/ijms25147517 39062758 PMC11276856

[8] Altinoz MA Ozpinar A Alturfan EE Elmaci I . Vinorelbine's anti-tumor actions may depend on the mitotic apoptosis, autophagy and inflammation: hypotheses with implications for chemo-immunotherapy of advanced cancers and pediatric gliomas. J Chemother. (2018) 30:203–12. doi: 10.1080/1120009x.2018.1487149 30025492

[9] Capasso A . Vinorelbine in cancer therapy. Curr Drug Targets. (2012) 13:1065–71. doi: 10.2174/138945012802009017 22594474

[10] Rugo HS Tolaney SM Loirat D Punie K Bardia A Hurvitz SA . Safety analyses from the phase 3 ASCENT trial of sacituzumab govitecan in metastatic triple-negative breast cancer. NPJ Breast Cancer. (2022) 8:98. doi: 10.1038/s41523-022-00467-1 36038616 PMC9424318

[11] Thomas-Schoemann A Lemare F Mongaret C Bermudez E Chéreau C Nicco C . Bystander effect of vinorelbine alters antitumor immune response. Int J Cancer. (2011) 129:1511–8. doi: 10.1002/ijc.25813 21128224

[12] Bardia A Jhaveri K Im SA Pernas S De Laurentiis M Wang S . Datopotamab deruxtecan versus chemotherapy in previously treated inoperable/metastatic hormone receptor-positive human epidermal growth factor receptor 2-negative breast cancer: primary results from TROPION-breast01. J Clin Oncol. (2025) 43:285–96. doi: 10.1200/jco.24.00920 39265124 PMC11771365

[13] Liu C Li X Huang Q Zhang M Lei T Wang F . Single-cell RNA-sequencing reveals radiochemotherapy-induced innate immune activation and MHC-II upregulation in cervical cancer. Signal Transduct Tgt Ther. (2023) 8:44. doi: 10.1038/s41392-022-01264-9 36710358 PMC9884664

[14] Ou Z Lin S Qiu J Ding W Ren P Chen D . Single-nucleus RNA sequencing and spatial transcriptomics reveal the immunological microenvironment of cervical squamous cell carcinoma. Adv Sci Weinh. (2022) 9:e2203040. doi: 10.1101/2021.12.23.473944 35986392 PMC9561780

[15] Liu C Zhang M Yan X Ni Y Gong Y Wang C . Single-cell dissection of cellular and molecular features underlying human cervical squamous cell carcinoma initiation and progression. Sci Adv. (2023) 9:eadd8977. doi: 10.1126/sciadv.add8977 36706185 PMC9882988

[16] Bassiouni R Idowu MO Gibbs LD Robila V Grizzard PJ Webb MG . Spatial transcriptomic analysis of a diverse patient cohort reveals a conserved architecture in triple-negative breast cancer. Cancer Res. (2023) 83:34–48. doi: 10.1158/0008-5472.can-22-2682 36283023 PMC9812886

[17] Ritchie ME Phipson B Wu D Hu Y Law CW Shi W . limma powers differential expression analyses for RNA-sequencing and microarray studies. Nucleic Acids Res. (2015) 43:e47. doi: 10.1093/nar/gkv007 25605792 PMC4402510

[18] Gustavsson EK Zhang D Reynolds RH Garcia-Ruiz S Ryten M . ggtranscript: an R package for the visualization and interpretation of transcript isoforms using ggplot2. Bioinformatics. (2022) 38:3844–6. doi: 10.1093/bioinformatics/btac409 35751589 PMC9344834

[19] Gu Z . Complex heatmap visualization. Imeta. (2022) 1:e43. doi: 10.1002/imt2.43 38868715 PMC10989952

[20] Miyake M Sakamoto J Kondo H Iwakura A Doi H Tamura T . Forty-year survival after Glenn procedure without Fontan procedure in patients with single ventricle. Eur J Cardiothorac Surg. (2023) 63:ezac528. doi: 10.1093/ejcts/ezac528 36322816 PMC9942551

[21] Yu G Wang LG Han Y He QY . clusterProfiler: an R package for comparing biological themes among gene clusters. Omics. (2012) 16:284–7. doi: 10.1089/omi.2011.0118 22455463 PMC3339379

[22] Yao Q Wu Q Xu X Xing Y Liang J Lin Q . Resveratrol ameliorates systemic sclerosis via suppression of fibrosis and inflammation through activation of SIRT1/mTOR signaling. Drug Des Devel Ther. (2020) 14:5337–48. doi: 10.2147/dddt.s281209 33293795 PMC7719308

[23] Liu P Xu H Shi Y Deng L Chen X . Potential molecular mechanisms of plantain in the treatment of gout and hyperuricemia based on network pharmacology. Evid Based Complement Alternat Med. (2020) 2020:3023127. doi: 10.1155/2020/3023127 33149752 PMC7603577

[24] Lei J Qu T Cha L Tian L Qiu F Guo W . Clinicopathological characteristics of pheochromocytoma/paraganglioma and screening of prognostic markers. J Surg Oncol. (2023) 128:510–8. doi: 10.1002/jso.27358 37272486

[25] Li Y Lu F Yin Y . Applying logistic LASSO regression for the diagnosis of atypical Crohn's disease. Sci Rep. (2022) 12:11340. doi: 10.1038/s41598-022-15609-5 35790774 PMC9256608

[26] Jiang YC Xu QT Wang HB Ren SY Zhang Y . A novel prognostic signature related to programmed cell death in osteosarcoma. Front Immunol. (2024) 15:1427661. doi: 10.3389/fimmu.2024.1427661 39015570 PMC11250594

[27] Zhao P Zhen H Zhao H Huang Y Cao B . Identification of hub genes and potential molecular mechanisms related to radiotherapy sensitivity in rectal cancer based on multiple datasets. J Transl Med. (2023) 21:176. doi: 10.1186/s12967-023-04029-2 36879254 PMC9987056

[28] Parvandeh S Yeh HW Paulus MP McKinney BA . Consensus features nested cross-validation. Bioinf Oxford England. (2020) 36:3093–8. doi: 10.1093/bioinformatics/btaa046 31985777 PMC7776094

[29] Sachs MC . plotROC: a tool for plotting ROC curves. J Stat Softw. (2017) 79:2. doi: 10.18637/jss.v079.c02 30686944 PMC6347406

[30] Zhang X Zeng B Zhu H Ma R Yuan P Chen Z . Role of glycosphingolipid biosynthesis coregulators in Malignant progression of thymoma. Int J Biol Sci. (2023) 19:4442–56. doi: 10.7150/ijbs.83468 37781041 PMC10535712

[31] Newman AM Liu CL Green MR Gentles AJ Feng W Xu Y . Robust enumeration of cell subsets from tissue expression profiles. Nat Methods. (2015) 12:453–7. doi: 10.1038/nmeth.3337 25822800 PMC4739640

[32] Chen B Khodadoust MS Liu CL Newman AM Alizadeh AA . Profiling tumor infiltrating immune cells with CIBERSORT. Methods Mol Biol. (2018) 1711:243–59. doi: 10.1007/978-1-4939-7493-1_12 29344893 PMC5895181

[33] Xiang T Wei Z Ye C Liu G . Prognostic impact and immunotherapeutic implications of NETosis-related gene signature in gastric cancer patients. J Cell Mol Med. (2024) 28:e18087. doi: 10.1111/jcmm.18087 38146607 PMC10902305

[34] Yoshihara K Shahmoradgoli M Martínez E Vegesna R Kim H Torres-Garcia W . Inferring tumour purity and stromal and immune cell admixture from expression data. Nat Commun. (2013) 4:2612. doi: 10.1038/ncomms3612 24113773 PMC3826632

[35] Mayakonda A Lin DC Assenov Y Plass C Koeffler HP . Maftools: efficient and comprehensive analysis of somatic variants in cancer. Genome Res. (2018) 28:1747–56. doi: 10.1101/gr.239244.118 30341162 PMC6211645

[36] Geeleher P Cox N Huang RS . pRRophetic: an R package for prediction of clinical chemotherapeutic response from tumor gene expression levels. PloS One. (2014) 9:e107468. doi: 10.1371/journal.pone.0107468 25229481 PMC4167990

[37] Satija R Farrell JA Gennert D Schier AF Regev A . Spatial reconstruction of single-cell gene expression data. Nat Biotechnol. (2015) 33:495–502. doi: 10.1038/nbt.3192 25867923 PMC4430369

[38] Wolf FA Angerer P Theis FJ . SCANPY: large-scale single-cell gene expression data analysis. Genome Biol. (2018) 19:15. doi: 10.1186/s13059-017-1382-0 29409532 PMC5802054

[39] Yang S Gu C Miao X Zuo H Xu W Zhang Y . Single-cell and spatial transcriptome profiling identifies the immunosuppressive spatial niche in KRAS-mutant colorectal cancer. J Immunother Cancer. (2025) 13:e013763. doi: 10.1136/jitc-2025-013763 41475845 PMC12766835

[40] Chen Z Dai Y Gao F Liu J He J Zhang L . Integrative analysis of crosstalk genes and diagnostic biomarkers in lupus-associated osteoporosis. Int J Immunopathol Pharmacol. (2025) 39:3946320251331842. doi: 10.1177/03946320251331842 40298129 PMC12041714

[41] Luo J Deng M Zhang X Sun X . ESICCC as a systematic computational framework for evaluation, selection, and integration of cell-cell communication inference methods. Genome Res. (2023) 33:1788–805. doi: 10.1101/gr.278001.123 37827697 PMC10691505

[42] Trapnell C Cacchiarelli D Grimsby J Pokharel P Li S Morse M . The dynamics and regulators of cell fate decisions are revealed by pseudotemporal ordering of single cells. Nat Biotechnol. (2014) 32:381–6. doi: 10.1038/nbt.2859 24658644 PMC4122333

[43] Pao YS Liao KJ Shiau YC Chao MH Li MC Lin LM . KIF2C promotes paclitaxel resistance by depolymerizing polyglutamylated microtubules. Dev Cell. (2025) 60:2097–113.e8. doi: 10.1016/j.devcel.2025.03.004 40157365

[44] Abouzeid HA Kassem L Liu X Abuelhana A . Paclitaxel resistance in breast cancer: current challenges and recent advanced therapeutic strategies. Cancer Treat Res Commun. (2025) 43:100918. doi: 10.1016/j.ctarc.2025.100918 40215760

[45] Li J Chen M Tong M Cao Q . TUBA1C orchestrates the immunosuppressive tumor microenvironment and resistance to immune checkpoint blockade in clear cell renal cell carcinoma. Front Immunol. (2024) 15:1457691. doi: 10.3389/fimmu.2024.1457691 39301023 PMC11410638

[46] Zhang Y Zhong F Liu L . Single-cell transcriptional atlas of tumor-associated macrophages in breast cancer. Breast Cancer Res. (2024) 26:129. doi: 10.1186/s13058-024-01887-6 39232806 PMC11373130

[47] Hu J Han C Zhong J Liu H Liu R Luo W . Dynamic network biomarker of pre-exhausted CD8(+) T cells contributed to T cell exhaustion in colorectal cancer. Front Immunol. (2021) 12:691142. doi: 10.3389/fimmu.2021.691142 34434188 PMC8381053

[48] Tutt ANJ Garber JE Kaufman B Viale G Fumagalli D Rastogi P . Adjuvant olaparib for patients with BRCA1- or BRCA2-mutated breast cancer. N Engl J Med. (2021) 384:2394–405. doi: 10.1007/s12282-023-01451-8 34081848 PMC9126186

[49] Metcalfe K Huzarski T Gronwald J Kotsopoulos J Kim R Moller P . Risk-reducing mastectomy and breast cancer mortality in women with a BRCA1 or BRCA2 pathogenic variant: an international analysis. Br J Cancer. (2024) 130:269–74. doi: 10.1038/s41416-023-02503-8 38030749 PMC10803363

[50] Saleem M Ghazali MB Wahab M Yusoff NM Mahsin H Seng CE . The BRCA1 and BRCA2 genes in early-onset breast cancer patients. Adv Exp Med Biol. (2020) 1292:1–12. doi: 10.1007/5584_2018_147 29687286

[51] Chu DT Vu NSM Vu TH Vu TD Nguyen MH Singh V . The expression and mutation of BRCA1/2 genes in ovarian cancer: a global systematic study. Expert Rev Mol Diagn. (2023) 23:53–61. doi: 10.1080/14737159.2023.2168190 36634123

[52] Zhong Y Li F Zhang S Yang Z Ren X Cao X . Syndecan-1 as an immunogene in triple-negative breast cancer: regulation tumor-infiltrating lymphocyte in the tumor microenviroment and EMT by TGFb1/Smad pathway. Cancer Cell Int. (2023) 23:76. doi: 10.1186/s12935-023-02917-7 37069585 PMC10111802

[53] Li Q Yuan H Zhao G Zhang J Li S Gong D . ZNF32 prevents the activation of cancer-associated fibroblasts through negative regulation of TGFB1 transcription in breast cancer. FASEB J. (2023) 37:e22837. doi: 10.1096/fj.202201801R 36934389

[54] Wang L Wang H Zhu M Ni X Sun L Wang W . Platelet-derived TGF-β1 induces functional reprogramming of myeloid-derived suppressor cells in immune thrombocytopenia. Blood. (2024) 144:99–112. doi: 10.1182/blood-2023-182619 38574321

[55] Nalio Ramos R Missolo-Koussou Y Gerber-Ferder Y Bromley CP Bugatti M Núñez NG . Tissue-resident FOLR2(+) macrophages associate with CD8(+) T cell infiltration in human breast cancer. Cell. (2022) 185:1189–1207.e25. doi: 10.1016/j.cell.2022.02.021 35325594

[56] Seung E Xing Z Wu L Rao E Cortez-Retamozo V Ospina B . A trispecific antibody targeting HER2 and T cells inhibits breast cancer growth via CD4 cells. Nature. (2022) 603:328–34. doi: 10.1038/s41586-022-04439-0 35197632

[57] Zhu Q Han Y He Y Meng P Fu Y Yang H . Quercetin inhibits neuronal ferroptosis and promotes immune response by targeting lipid metabolism-related gene PTGS2 to alleviate breast cancer-related depression. Phytomedicine. (2024) 130:155560. doi: 10.1016/j.phymed.2024.155560 38815404

[58] Balamurugan K Poria DK Sehareen SW Krishnamurthy S Tang W McKennett L . Stabilization of E-cadherin adhesions by COX-2/GSK3β signaling is a targetable pathway in metastatic breast cancer. JCI Insight. (2023) 8:e156057. doi: 10.1172/jci.insight.156057 36757813 PMC10070121

[59] Wang Z Wang X Gao Y Wang Y Xu M Han Q . IL-7R gene variants are associated with breast cancer susceptibility in Chinese Han women. Int Immunopharmacol. (2020) 86:106756. doi: 10.1016/j.intimp.2020.106756 32659700

[60] Coppola C Hopkins B Huhn S Du Z Huang Z Kelly WJ . Investigation of the impact from IL-2, IL-7, and IL-15 on the growth and signaling of activated CD4(+) T cells. Int J Mol Sci. (2020) 21:7814. doi: 10.3390/ijms21217814 33105566 PMC7659484

[61] Pascal M Bax HJ Bergmann C Bianchini R Castells M Chauhan J . Granulocytes and mast cells in AllergoOncology-bridging allergy to cancer: an EAACI position paper. Allergy. (2024) 79:2319–45. doi: 10.1111/all.16246 39036854

[62] Gong Y Wu J Hu Y . m(6)A epitranscriptomic regulation of KRAS by METTL3 promotes EMT and stromal remodeling through TGF-β/SMAD signaling in cervical cancer. Cancer Gene Ther. (2026) 33:198–211. doi: 10.1038/s41417-025-00993-7 41484386

[63] Mariathasan S Turley SJ Nickles D Castiglioni A Yuen K Wang Y . TGFβ attenuates tumour response to PD-L1 blockade by contributing to exclusion of T cells. Nature. (2018) 554:544–8. doi: 10.1038/nature25501 29443960 PMC6028240

[64] Al-Omar A Asadi M Mert U Muftuoglu C Karakus HS Goksel T . Effects of vinorelbine on M2 macrophages in non-small cell lung cancer. Int J Mol Sci. (2025) 26:2252. doi: 10.3390/ijms26052252 40076874 PMC11900078

[65] Zheng M Li Y Hu W Cai J Zhang H Yang Z . Ruhe Sanjie Tablet ameliorates depression-associated mammary gland hyperplasia by modulating estrogen receptor signaling and CDK2. J Ethnopharmacol. (2026) 359:121025. doi: 10.1016/j.jep.2025.121025 41380859

[66] Majumder C Manna A Halder S Roy S Mandal SC Jana K . Indole-alkaloid-rich fraction of Ervatamia coronaria leaf extract regresses breast cancer by inducing apoptotic cell death. Bio/Technol Rep Amst. (2026) 49:e00937. doi: 10.1016/j.btre.2025.e00937 41439032 PMC12720107

[67] Song D Wei Y Zhao X Ning D . ECRG4 suppressed the progression of breast cancer via modulating NFIC/PTEN and SHP2/PI3K/SP1 signaling. Eur J Med Res. (2026). doi: 10.1186/s40001-026-04009-4 41664108

[68] Xia T Dai XY Sang MY Zhang X Xu F Wu J . IGF2BP2 drives cell cycle progression in triple-negative breast cancer by recruiting EIF4A1 to promote the m6A-modified CDK6 translation initiation process. Adv Sci Weinh. (2024) 11:e2305142. doi: 10.1002/advs.202305142 37983610 PMC10767445

[69] Ali MM Itoh Y Badji AMP Gallant S Tsirigoti C Bai Y . TGFβ signaling promotes cell cycle progression and resistance to the CDK4/6 inhibitor palbociclib through SOX4 transcriptional modulation in breast cancer cells. Cell Death Dis. (2026) 17:209. doi: 10.1038/s41419-026-08435-4 41639049 PMC12895049

