# Supplementary material for: Identification of potential vinorelbine-associated prognostic genes in breast cancer through integrative bioinformatics and experimental validation
Source: Front Oncol. 2026 Jun 26;16:1855523. doi: 10.3389/fonc.2026.1855523 (PMC13349924; doi:10.3389/fonc.2026.1855523)
Supplement: Supplementary file 1 [file DataSheet1.zip › Supplementary Material/Supplementary Table 2(Revised).docx]

| **Primer** | **Sequence** | |
| --- | --- | --- |
| TGFB1 F | CGTGGAAATCAACGCTCCAC | |
| TGFB1 R | CCACGTAGTAGACGATGGGC | |
| IL7 F | TGCTGCACATTTGTGGCTTC |  |
| IL7 R | ACCAGGATCAGTGGTTGCTG |  |
| PTGS2 F | TGAGTGGGGTGATGAGCAAC |  |
| PTGS2 R | TTCAGAGGCAATGCGGTTCT |  |
| BRCA1 F | ACCTTGGAACTGTGAGAACTCT |  |
| BRCA1 R | TCTTGATCTCCCACACTGCAATA |  |
| TUBA1C   F | GAGGTTGGGGCAGATAGTGC |  |
| TUBA1C   R | AGAGCACACACAAAGACAAAGA |  |
| TUBA1B   F | CTGCCATTGCCACCATCAAG |  |
| TUBA1B   R | GGGGCTCAAGGAATGGACTTA |  |
| TUBB2B   F | GGCATGGACGAGATGGAGTT |  |
| TUBB2B   R | AGCTTTCCCTAACCTGCTTGG |  |
| XRCC1   F | CATGCCTGGTGCTTTCGAG |  |
| XRCC1   R | GCTCTTGTAGAGGACTGGGG |  |
| Internal reference M-GAPDH F | TGTGTCCGTCGTGGATCTGA |  |
| Internal reference M-GAPDH R | GAGTTGCTGTTGAAGTCGCA |  |
