# Supplementary figures and images for: Identification of potential vinorelbine-associated prognostic genes in breast cancer through integrative bioinformatics and experimental validation
Source: Front Oncol. 2026 Jun 26;16:1855523. doi: 10.3389/fonc.2026.1855523 (PMC13349924; doi:10.3389/fonc.2026.1855523)

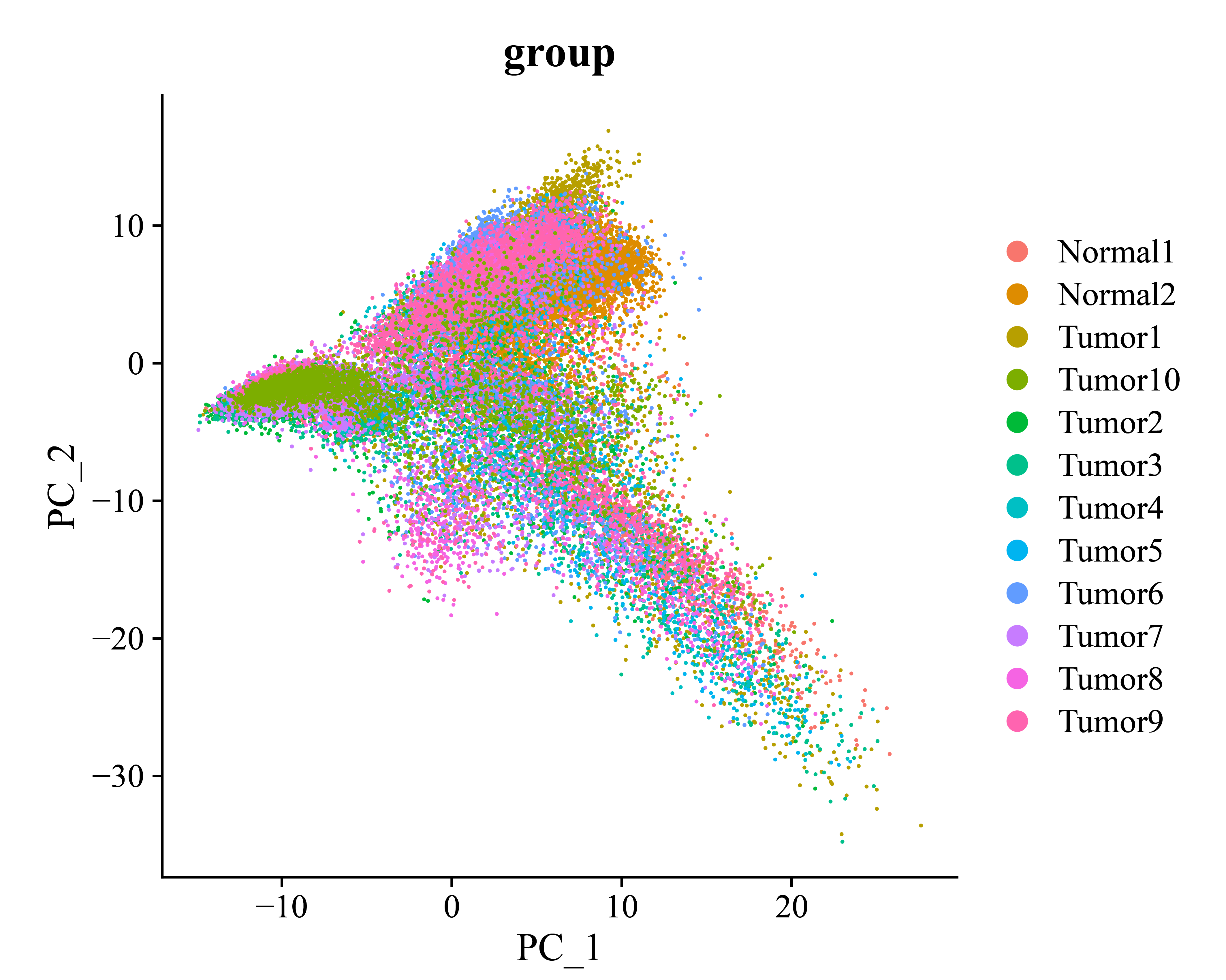

Supplement: Supplementary file 1 [file DataSheet1.zip › Supplementary Material/Supplementary Figure 1.tif]

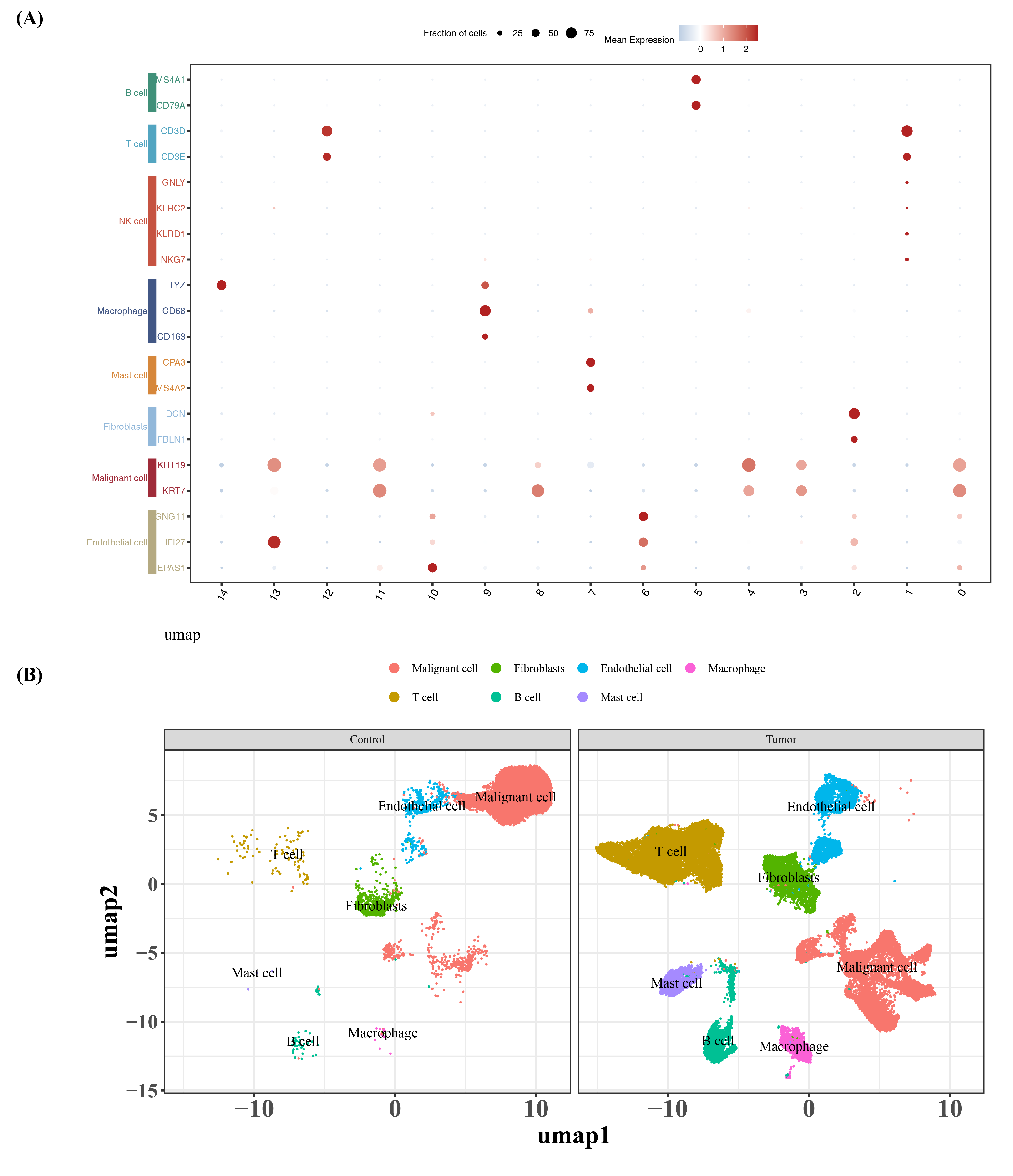

Supplement: Supplementary file 1 [file DataSheet1.zip › Supplementary Material/Supplementary Figure 2.tif]

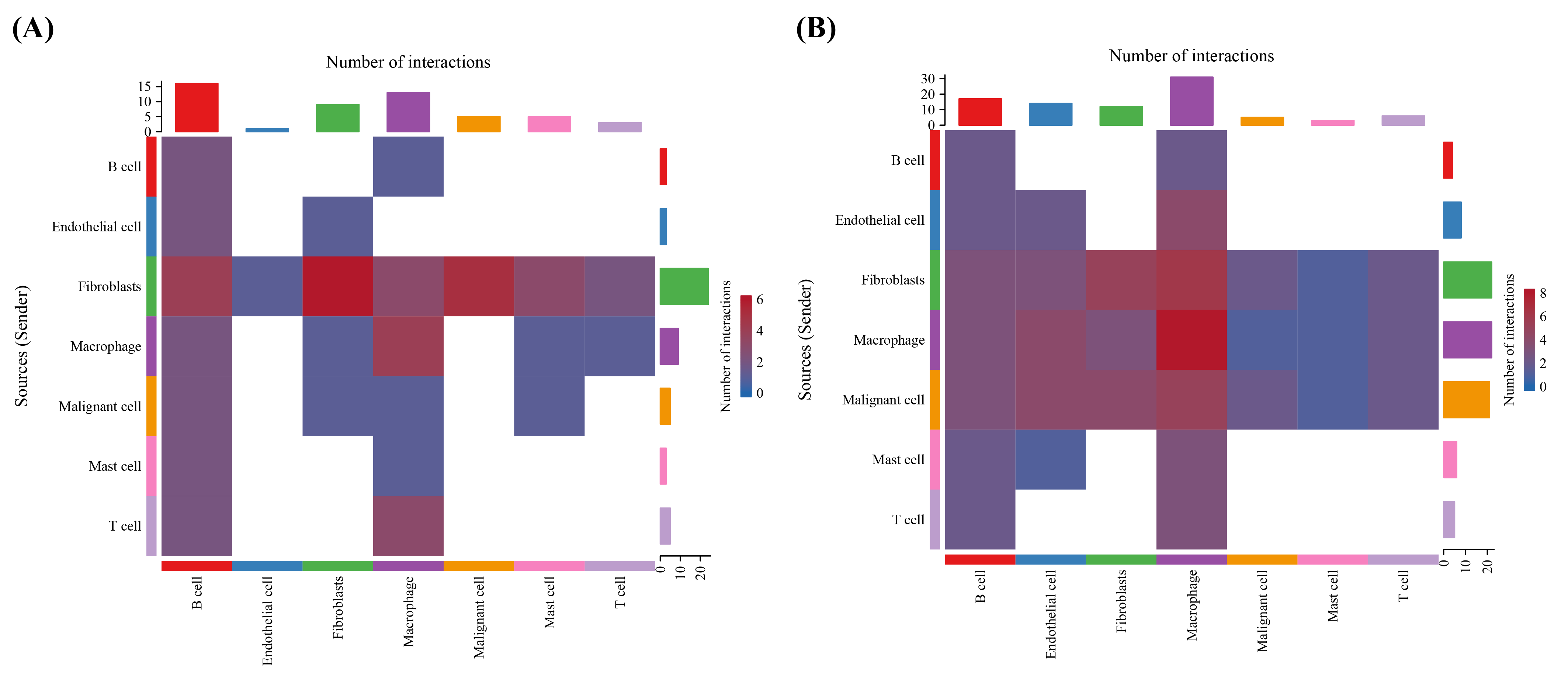

Supplement: Supplementary file 1 [file DataSheet1.zip › Supplementary Material/Supplementary Figure 3.tif]

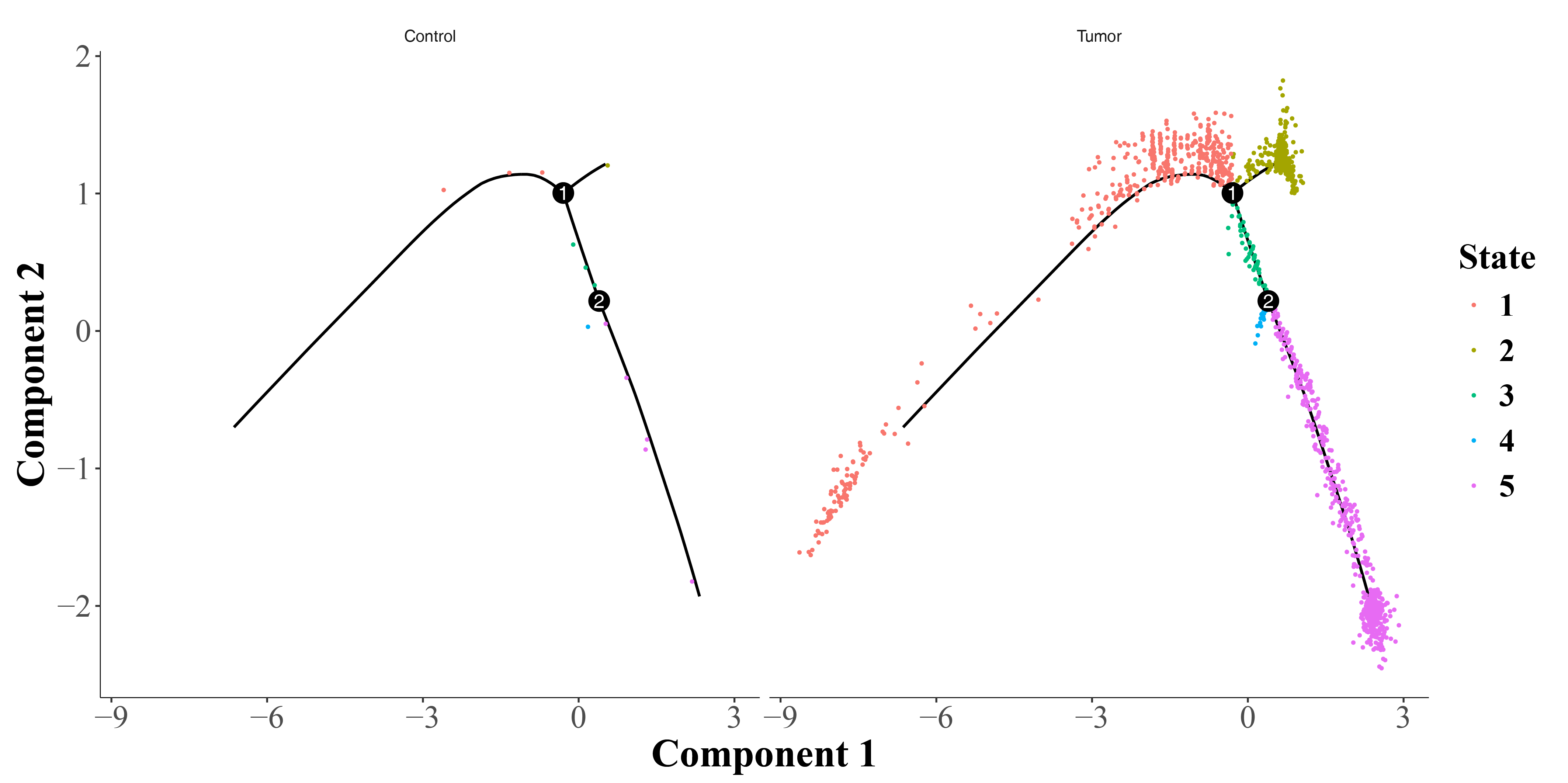

Supplement: Supplementary file 1 [file DataSheet1.zip › Supplementary Material/Supplementary Figure 4.tif]

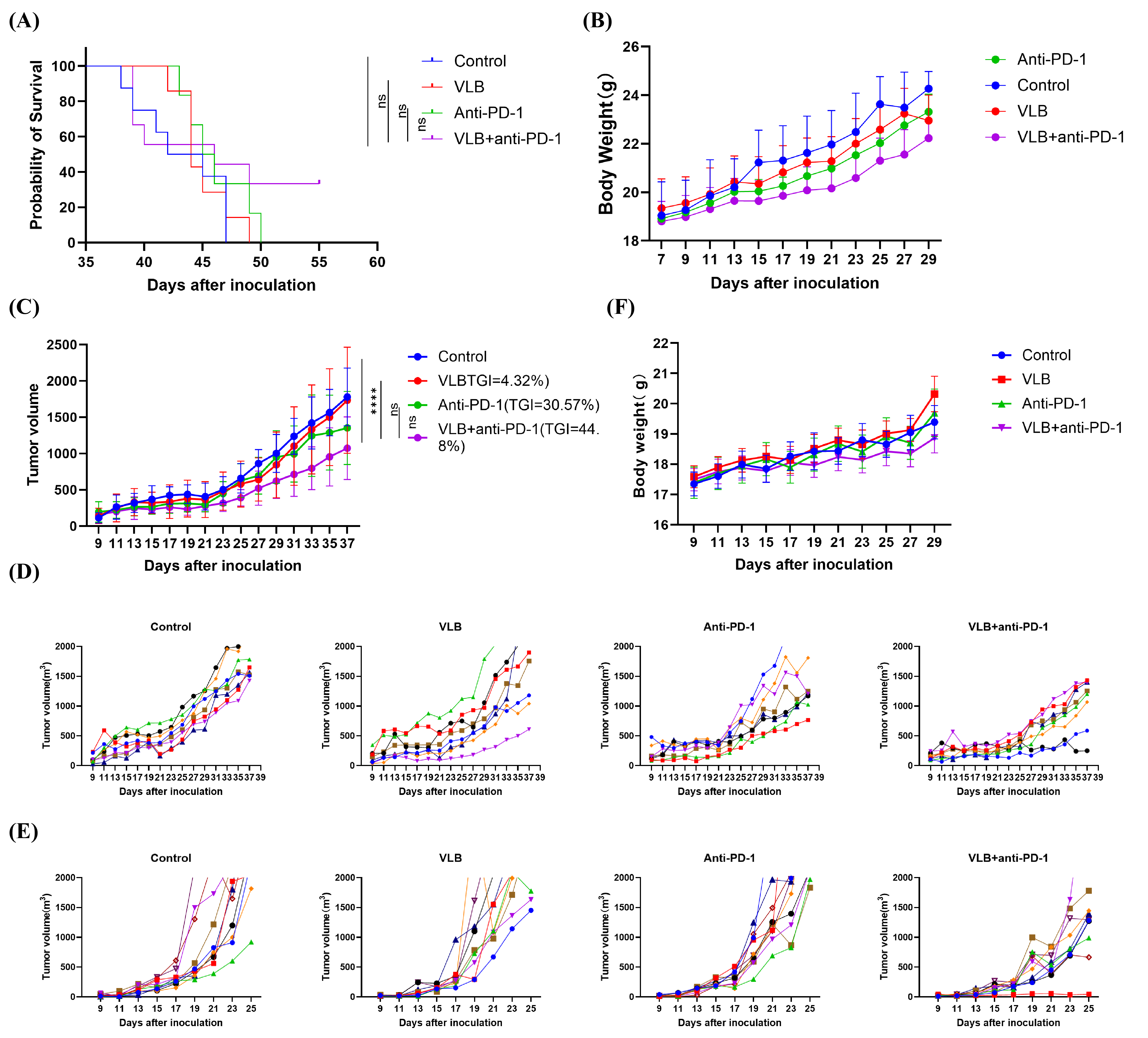

Supplement: Supplementary file 1 [file DataSheet1.zip › Supplementary Material/Supplementary Figure 5.tif]

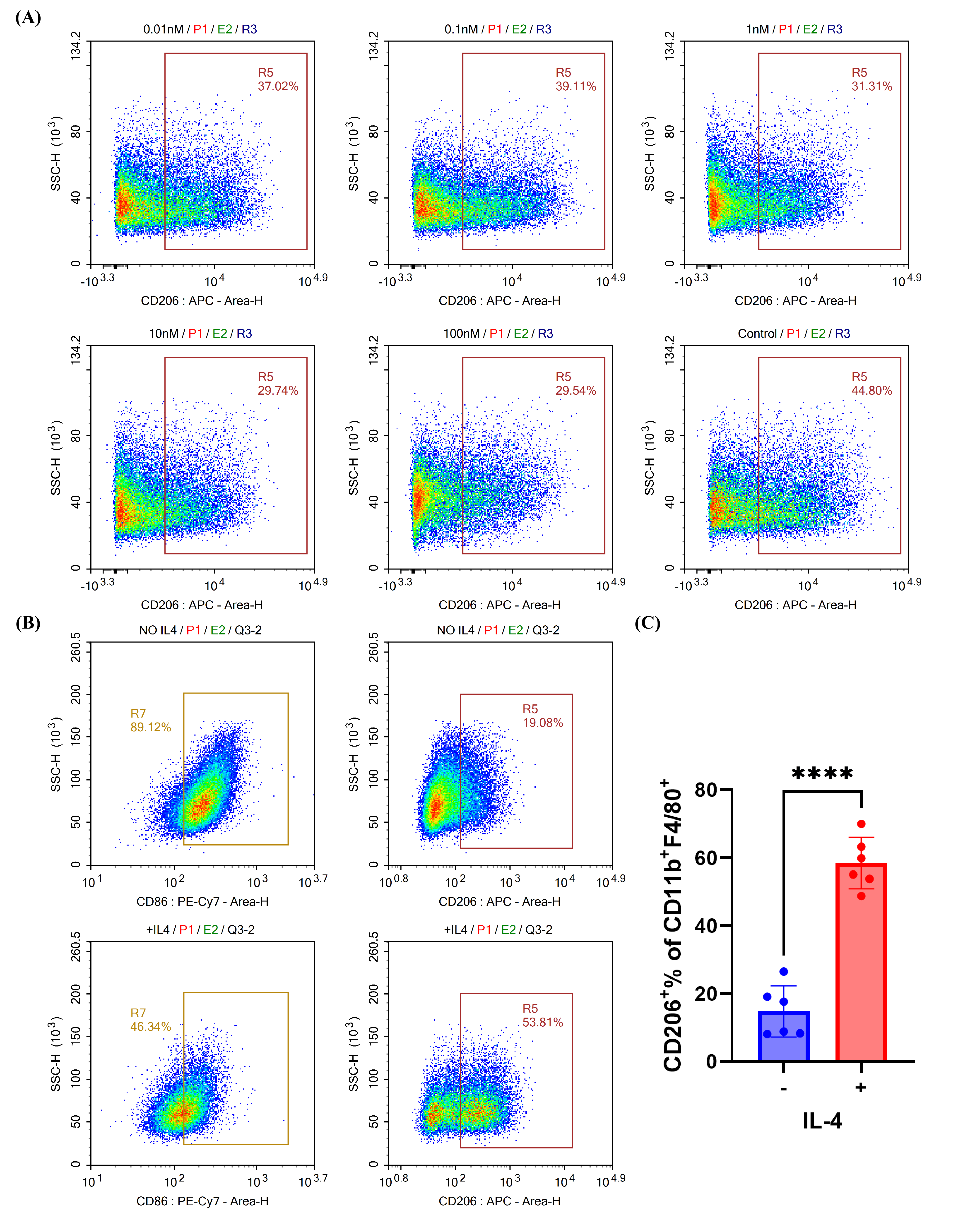

Supplement: Supplementary file 1 [file DataSheet1.zip › Supplementary Material/Supplementary Figure 6.tif]
